# Selective Interleukin-6 Trans-Signaling Blockade Is More Effective Than Panantagonism in Reperfused Myocardial Infarction

**DOI:** 10.1016/j.jacbts.2021.01.013

**Published:** 2021-04-07

**Authors:** Marc Jonathan George, Nur Hayati Jasmin, Valerie Taylor Cummings, Angela Richard-Loendt, Francesca Launchbury, Kevin Woollard, Tabitha Turner-Stokes, Ana Isabel Garcia Diaz, Mark Lythgoe, Daniel James Stuckey, Aroon Dinesh Hingorani, Derek William Gilroy

**Affiliations:** a Department of Clinical Pharmacology, Division of Medicine, University College London, London, United Kingdom; b Department of Clinical Pharmacology, University College London Hospitals NHS Foundation Trust, London, United Kingdom; c Centre for Advanced Biomedical Imaging (CABI), Division of Medicine, University College London, London, United Kingdom; d Department of Neuropathology, University College London Hospitals NHS Foundation Trust, London, United Kingdom; e UCL IQPath, Institute of Neurology, University College London, London, United Kingdom; f Immunology and Inflammation, Faculty of Medicine, Imperial College London, London, United Kingdom; g Centre for Translational Genomics, Institute of Cardiovascular Science, University College London, London, United Kingdom

**Keywords:** inflammation, interleukin-6, myocardial infarction, reperfusion, AAR, area at risk, Ab, antibody, c-caspase-3, cleaved caspase-3, CCL, C-C motif chemokine ligand, CMR, cardiac magnetic resonance, CXCL, C-X-C motif ligand, ICAM-1, intercellular adhesion molecule 1, IL, interleukin, IS, infarct size, LGE, late-gadolinium enhancement, LVEF, left ventricular ejection fraction, MHC, major histocompatibility complex, MI, myocardial infarction, NSTEMI, non–ST-segment-elevation MI, RCAEC, rat coronary artery endothelial cell, sIL-6R, soluble IL-6 receptor, STEMI, ST-segment-elevation MI, TCZ, tocilizumab, Trop-T, troponin T

## Abstract

Interleukin (IL)-6 is an emerging therapeutic target in myocardial infarction (MI). IL-6 has 2 distinct signaling pathways: trans-signaling, which mediates inflammation, and classic signaling, which also has anti-inflammatory effects. The novel recombinant fusion protein sgp130Fc achieves exclusive trans-signaling blockade, whereas anti–IL-6 antibodies (Abs) result in panantagonism. In a rat model of reperfused MI, sgp130Fc, but not anti–IL-6-Ab, attenuated neutrophil and macrophage infiltration into the myocardium, reduced infarct size, and preserved cardiac function 28 days after MI. These data demonstrate the efficacy of exclusive IL-6 trans-signaling blockade and support further investigation of sgp130Fc as a potential novel therapy in MI.

## Highlights

• The inflammatory cytokine IL-6 is an emerging therapeutic target in MI.
• In a rat model of reperfused MI, IL-6 is rapidly up-regulated and remains elevated for 7 days.
• The novel recombinant protein sgp130Fc achieves selective IL-6 trans-signaling blockade, whereas anti–IL-6 Ab blocks both trans- and classic signaling.
• sgp130Fc but not anti–IL-6-Ab attenuated neutrophil and macrophage infiltration into the myocardium, reduced infarct size, and preserved cardiac function 28 days after MI.
• Because sgp130Fc has already entered clinical phase II testing (for other indications), it could also be rapidly evaluated in humans as a potential novel therapy in MI.

## Visual Abstract

**Figure:**
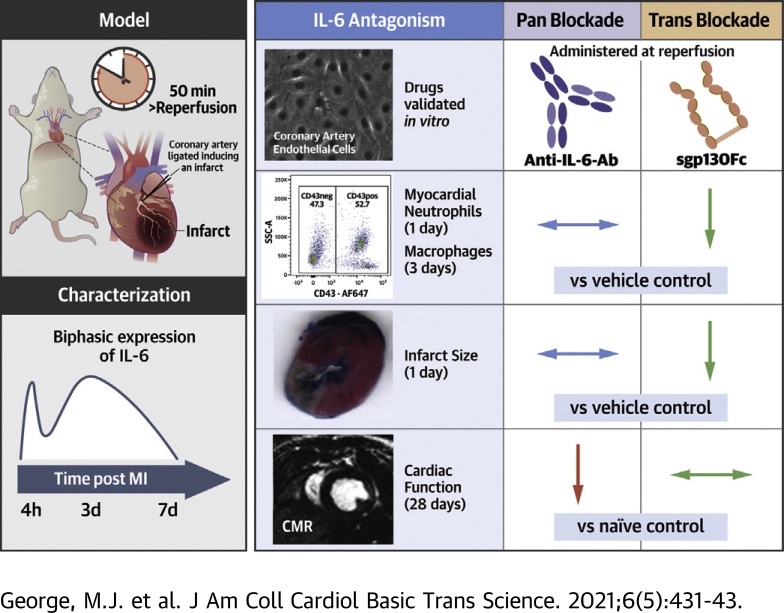

Despite modern therapies, myocardial infarction (MI) remains a leading cause of heart failure, a syndrome associated with significant morbidity and mortality (1). Therefore, there is a pressing need for new therapeutic approaches, which can reduce the development of this debilitating condition after MI. Several strands of evidence have suggested that targeting the inflammatory cytokine interleukin-6 (IL-6) may be such an approach.

First, data from multiple large clinical studies have demonstrated a strong association between IL-6 and adverse outcomes after MI (2). Second, IL-6 plays a central role in coordinating the acute inflammatory response (3, 4, 5) and contributes directly to neutrophil-mediated myocyte necrosis (6). Third, its signaling can lead to excessive fibrosis and adverse remodeling, culminating in reduced cardiac function (7,8).

These and other data have prompted 2 clinical trials of IL-6 antagonism in MI. In the first, a single dose of the human anti–IL-6 receptor (IL-6R) monoclonal antibody (Ab) tocilizumab (TCZ) was administered to 117 patients presenting with non–ST-segment-elevation MI (NSTEMI), resulting in reduced C-reactive protein and troponin-T (Trop-T) (9). Crucially, these reductions and antiinflammatory effects of TCZ on the proteome (10) were seen only in the patients that underwent percutaneous coronary intervention. Therefore, targeting IL-6 is likely to be more effective in the context of reperfusion.

The follow-up ASSAIL-MI study (11) was performed in 199 patients with STEMI undergoing percutaneous coronary intervention (12). Preliminary data from the study have recently been presented showing that TCZ improved myocardial salvage index (12).

However, despite the promising results from these studies, it is unclear if targeting IL-6R is the best approach to modulating IL-6 in MI, given the complexities of its signaling and pleiotropic nature. IL-6 has 2 distinct signaling pathways termed classic and trans. Classic signaling is achieved by IL-6 binding to its membrane-bound α-receptor, which associates with a dimer of the receptor β-subunit glycoprotein 130 (gp130) to initiate intracellular signaling (13) (Supplemental Figure 1A). Classic signaling stimulates only cells that express membrane-bound IL-6R, including hepatocytes and leukocytes (13). Tissues may also respond to IL-6 via trans-signaling (Supplemental Figure 1B). This is achieved by IL-6 binding a circulating soluble form of the receptor (sIL-6R), and then the IL-6/sIL-6R complex subsequently binds to a dimer of membrane-bound gp130 (13). Because gp130 is ubiquitously expressed, this pathway enables IL-6 to stimulate all tissues regardless of their expression of membrane-bound IL-6R.

Crucially, whereas trans-signaling has proinflammatory effects facilitating leukocyte recruitment (13), classic signaling also has multiple well described anti-inflammatory effects (3,14). Drugs targeting IL-6 directly, or IL-6R, such as TCZ, achieve pan–IL-6 blockade, that is, they antagonize both pathways (15) (Supplemental Figure 1C). An alternate approach, to specifically target trans-signaling, is to use a novel recombinant form of gp130: sgp130Fc, a fusion protein of 2 gp130 molecules bound by IgG1-Fc that, compared with native sgp130, has 10–100 times higher affinity for the IL-6/sIL-6 complex (16). Because it has no affinity for free IL-6, it is an exclusive trans-signaling inhibitor (16) (Supplemental Figure 1D) and therefore leaves the anti-inflammatory effects of IL-6 classic signaling intact. sgp130Fc has been shown to reduce atherosclerosis in mice (17), but it has never been investigated in a model of MI.

In this paper, we present data demonstrating that sgp130Fc has greater anti-inflammatory effects than an anti–IL-6 antibody and is more effective in reducing infarct size and preserving cardiac function in an animal model of reperfused MI. This work identifies sgp130Fc as an eminently tractable potential novel therapy for MI.

## Methods

### Study design

The study was conducted in 3 stages. First, candidate anti–rat anti–IL-6 drugs were validated in vitro with the use of rat coronary artery endothelial cells (RCAECs). Second, a rat model was characterized to identify key time points in the IL-6 response after reperfused MI and to guide the design of subsequent therapeutic experiments. Third, the validated drugs were used in vivo to investigate the relative contribution of classic and trans-signaling to the acute inflammatory response and their therapeutic effects on infarct size and cardiac function.

### Rat coronary artery endothelial cell culture

Primary RCAECs isolated from 6–8-week-old Sprague-Dawley rats were used (CellBiologics, Chicago, Illinois). Confluent cells were used for experiments at P8 in 24-well plates (Corning Costar, Corning, New York). RCAECs were stimulated with recombinant IL-6 protein (Biolegend, San Diego, California) and recombinant rat soluble IL-6R (Sino Biological, Beijing, China) for 16 h. Soluble mediators in the supernate were measured by means of enzyme-linked immunosorbent assay and enzyme immunoassay. RCAEC surface expression of intercellular adhesion molecule (ICAM)-1 was measured by means of flow cytometry (antibody clone 1A29, conjugated to PE; Biolegend) and expressed as geometric mean florescence intensity. The antagonistic properties of several anti–IL-6 and anti–IL-6R antibodies were assessed. Further details are provided in the Supplemental Methods.

### Animal experiments

All investigators conformed to the guidelines from Directive 2010/63/EU of the European Parliament on the protection of animals used for scientific purposes, the Home Office Guidance on the Operation of the Animals (Scientific Procedures) Act 1986, and institutional guidelines. Approval was granted by the University College London Animal Welfare and Ethical Review Body and the Home Office (project license no. 70/8709, personal license under A(SP)A IEEABED33).

### Infarct model

Male Sprague-Dawley rats (Harlan, Indianapolis, Indiana) 7 to 8 weeks old and weighing 200–250 g were used. MI with reperfusion was induced as previously described (18). In brief, rats were anesthetized (2% isoflurane), and the left anterior descending coronary artery (LAD) was ligated for 50 min, followed by reperfusion. Sham procedures were performed in an identical manner but did not include ligation of the LAD (further details in Supplemental Methods).

### Flow cytometry

Inflammatory cells from circulating blood and myocardial digests were identified with the use of a panel of surface markers based on an approach previously described (19) (Supplemental Table 1). Cells were analyzed with the use of LSR Fortessa (Becton Dickinson [BD], Franklin Lakes, New Jersey). Gating for data acquisition was performed with the use of FACSDiva 8.0.1 (BD) and final analysis was performed with the use of FlowJo v.10.0.7 (BD). Gating strategy is detailed in Supplemental Figure 2 and Supplemental Table 2. CD11b was included as a measure of cell activation and expressed as geometric mean florescence intensity. Full methods for flow cytometry are detailed in the Supplemental Methods.

### Measurement of soluble mediators

Soluble mediators were measured in the supernate from myocardial digests and plasma using enzyme-linked immunosorbent assay and enzyme immunoassay kits. Kits were performed in accordance with the manufacturer’s instructions. MSD plates were read on a QuickPlex SQ 120 (Merck, Sharpe & Dohme, Kenilworth, New Jersey) and all others on a FluoStar Omega (BMG Labtech, Ortenberg, Germany), and normal curves of standards and extrapolated values were calculated with the use of Prism Version 6.0 (GraphPad, San Diego, California). The kits used are fully listed in the Supplemental Methods.

### Immunohistochemistry

Hearts were stained with antibodies against IL-6 (goat polyclonal; Bio-techne, Minneapolis, Minnesota), CD68 (rabbit polyclonal; Abcam, Cambridge, United Kingdom), and cleaved caspase-3 (c-caspase-3) (rabbit polyclonal; Cell Signaling, Danvers, Massachusetts) with the use of species-specific secondary antibodies (Dako, Glostrup, Denmark).

c-Caspase-3 expression of nonmyocytes was measured in the area of infarction in the apical slice. Positively stained cells were counted within a grid of 100 200-μm^2^ squares (total area: 4 mm^2^) and expressed as cells/mm^2^. Further details are provided in the Supplemental Methods.

### In vivo drug administration

In therapeutic experiments rats were randomized to receive 1 mL phosphate-buffered saline solution (PBS) (control and sham), or drugs reconstituted in 1 mL PBS 1 minute before reperfusion intravenously via the internal jugular vein. Drugs were administered in a blinded fashion. Rat sgp130Fc (R&D Systems, Minneapolis, Minnesota) was administered at a dose of 0.5 μg/g as previously described (17). Rabbit polyclonal anti–IL-6-Ab (AF506; R&D Systems) was administered at 0.1 μg/g. Because the intention was to provide a single bolus dose at reperfusion, this dose is 5× the previously reported daily intraperitoneal (IP) dose (20). However, because this antibody had not previously been administered in this manner, plasma measurements were taken to guide dosing (Supplemental Methods, Supplemental Figure 3). Based on these, a second dose of 0.1 μg/g was administered IP in 0.5 mL PBS 3 days after MI to ensure pan–IL-6 antagonism for at least 7 days after MI (total dose per rat 0.2 μg/g). PBS, rather than isotype controls, were used in the control group, after the relevant isotype controls were shown to be inactive in vitro (Supplemental Figure 4) and to avoid including additional groups of rats.

### Infarct size measurement

Infarct size and area at risk (AAR) in excised hearts was measured with Evans Blue and 2,3,5-triphenyl-tetrazolium-chloride dyes as previously described (21). Staining was performed on 6–8 parallel short-axis sections, and scans were analyzed with the use of ImageJ software (open source).

### Cardiac magnetic resonance imaging

Cardiac magnetic resonance (CMR) imaging was performed in rats 1 and 28 days of MI with a 9.4-T Varian MRI System (Palo Alto, California). Cardiac- and respiratory-gated cinematic (cine) CMR imaging was performed to measure cardiac structure and left ventricular ejection fraction (LVEF), followed by multislice inversion recovery images acquired 15 min after IP injection of 0.5 mmol/kg gadolinium (Magnevist; Bayer, Leverkusen, Germany) to measure late gadolinium enhancement (LGE). Enhancement on CMR images was presented as a percentage of the entire left ventricular myocardium. Full methods used for CMR are detailed in the Supplemental Methods.

### Statistical analysis

All statistical analyses were performed with the use of Prism v.6.0 (GraphPad, San Diego, California). Data are presented as mean ± SEM unless otherwise stated. Parametric tests were used after confirming gaussian distribution by applying the Shapiro-Wilk normality test. In experiments with multiple comparisons data were analyzed by means of 1-way analysis of variance with Dunnett correction for multiple pairwise comparisons to a control or Tukey correction for all pairwise comparisons as appropriate. Comparisons between 2 groups were made by means of unpaired Student *t*-tests and between 2 time points within a group by means of paired Student *t*-tests. A p value < 0.05 was considered to be statistically significant.

## Results

### Validation of anti-IL-6 drugs in vitro

**Figure 1:**
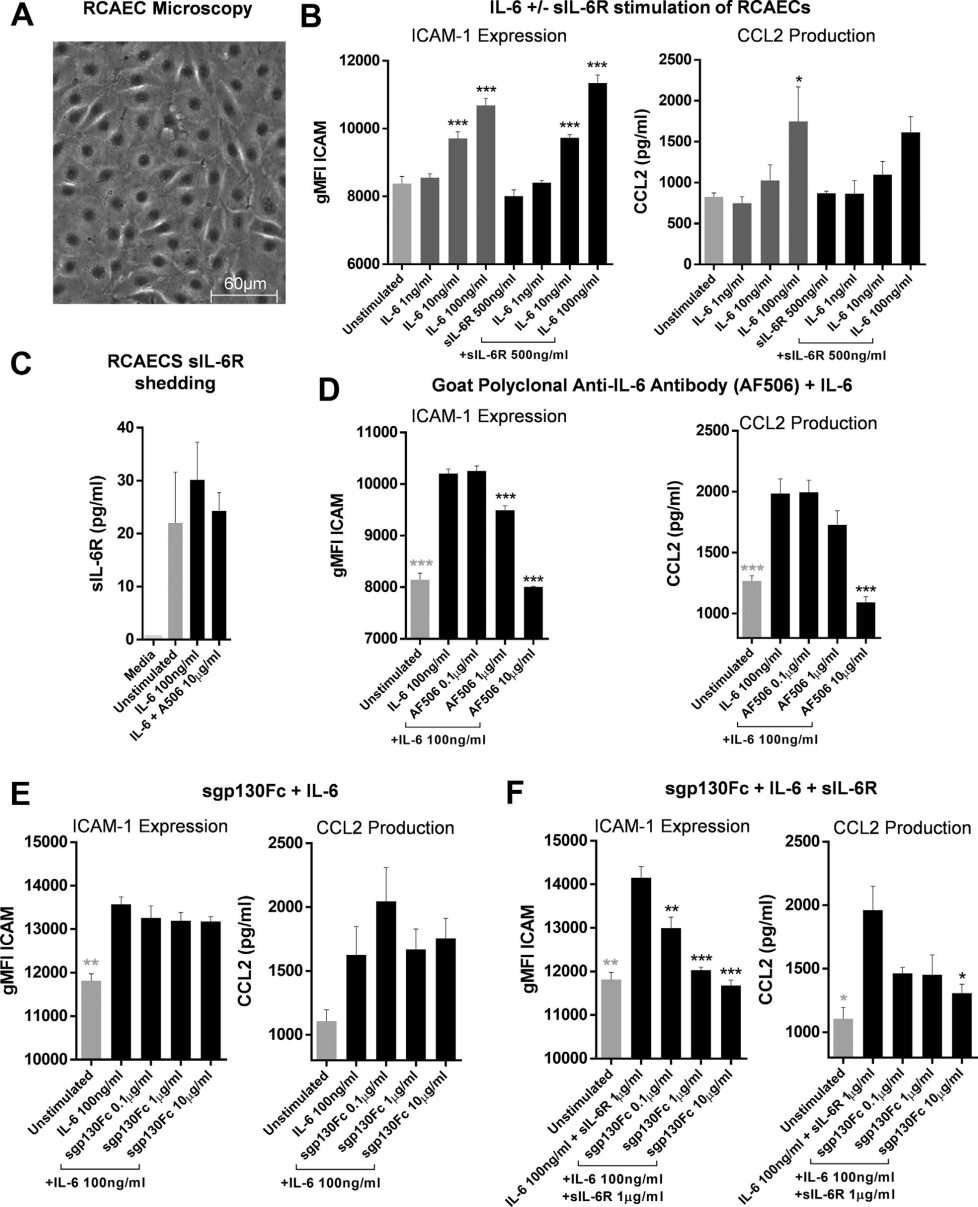
IL-6 Stimulation of RCAECs and In Vitro Antagonism of IL-6 Signaling With Anti–IL-6-Ab and sgp130Fc **(A)** RCAECs were seeded in 24-well plates at P8 and grown until confluent in serum-containing medium. Confluent cells were imaged with a light microscope to confirm endothelial cell morphology. **(B)** The cells were stimulated with 1, 10, or 100 ng/ml IL-6 ± 500 ng/ml sIL-6R in serum-free medium for 16 h. CCL2 was measured in the supernates by means of enzyme-linked immunosorbent assay (ELISA), and surface ICAM-1 expression was quantified by means of flow cytometry. **(C)** Shed sIL-6R was measured by means of ELISA in the supernate of RCAECs incubated for 16 h with and without 100 ng/ml IL-6. RCAECs were stimulated with 100 ng/ml IL-6 for 16 h with increasing concentrations of **(D)** anti–IL-6-Ab (AF506) and **(E)** sgp130Fc alone and **(F)** with sIL-6R. n = 3 (technical repeats), data shown as mean + SEM. Statistical significance was tested with the use of analysis of variance with multiple comparisons (comparing each condition with **[B, C]** unstimulated or **[D to F]**, 100 ng/ml IL-6; **black asterisks**) or unpaired Student's *t*-test (**D to F**, unstimulated vs. 10 ng/ml IL-6; **gray asterisks**). Multiplicity-adjusted p < 0.05 was considered to be significant. *∗*p < 0.05; ∗∗p < 0.01; ∗∗∗p < 0.001. ICAM = intercellular adhesion molecule; IL-6 = interleukin; RCAEC = rat coronary artery endothelial cell; sIL-6R = soluble IL-6 receptor.

RCAECs (Figure 1A) responded to IL-6 in a concentration-dependent manner by upregulating surface ICAM-1 expression and C-C motif chemokine ligand 2 (CCL2) production (Figure 1B). The addition of sIL-6R did not significantly affect the expression of either (Figure 1B). The cells shed sIL-6R, which was not influenced by IL-6 stimulation (Figure 1C).

**Figure 2:**
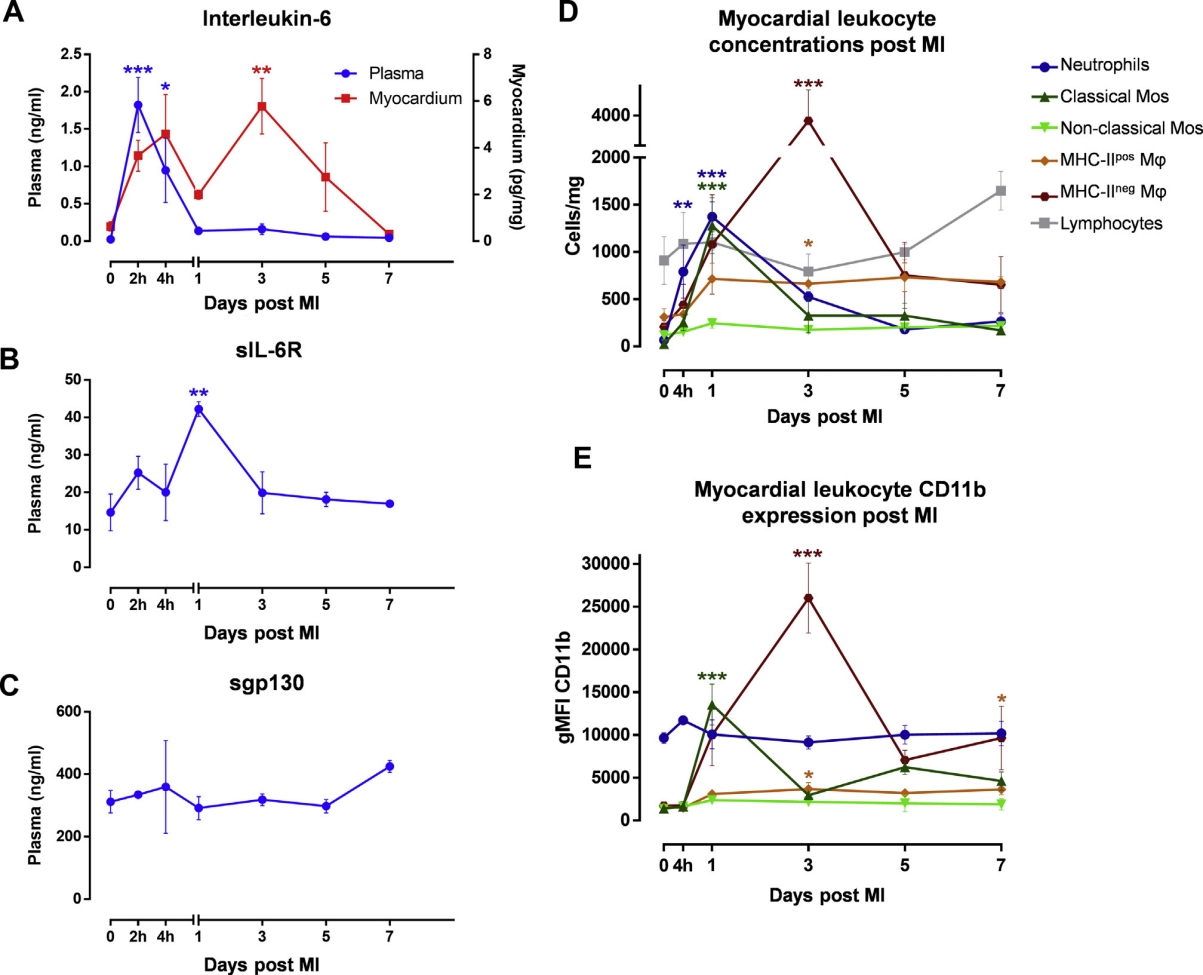
Temporal Profile of IL-6, sIL-6R, sgp130Fc, and Leukocytes After MI With Reperfusion Rats were subjected to surgical MI with 50 min of ischemia before reperfusion. **(A)** IL-6, **(B)** sIL-6R, and **(C)** sgp130Fc were measured by means of enzyme-linked immunosorbent assay in plasma **(blue)** ± supernates of heart digests **(red)** at 7 time points (0 h to 7 days after MI) (n = 3 to 4/group). **(D)** Leukocyte counts in the myocardium and **(E)** their CD11b expression were measured by means of flow cytometry at 6 time points (0 h to 7 days after MI) (n = 3 to 4/group). Statistical significance was tested by means of analysis of variance with multiple comparisons (comparing each time point with naive rats). Data presented as mean ± SEM. Multiplicity-adjusted p < 0.05 was considered to be significant. *∗*p < 0.05; ∗∗p < 0.01; ∗∗∗p < 0.001. MI = myocardial infarction; other abbreviations as in Figure 1.

Of a series of antibodies tested (Supplemental Table 3), all but 1 failed to antagonize IL-6 (Supplemental Figure 5). This was the goat polyclonal anti–IL-6 antibody AF506, which antagonized IL-6 in a concentration-dependent manner (Figure 1D). Therefore, AF506 (subsequently referred to as anti–IL-6-Ab) was used as an effective pan–IL-6 inhibitor.

Rat sgp130Fc had no direct effect on RCAEC ICAM-1 expression or CCL2 production (Supplemental Figure 5F). It also had no antagonistic effect on free IL-6 (Figure 1E). However, in the presence of sIL-6R, sgp130Fc antagonized IL-6 in a concentration-dependent manner (Figure 1E). These data demonstrated that sgp130Fc is an exclusive IL-6 trans-signaling inhibitor.

### Characterization of the inflammatory response after MI with reperfusion

The characterization provided novel insights into the temporal dynamics of IL-6 after MI. The concentration of IL-6 in plasma and myocardium after MI with reperfusion was biphasic (Figure 2A). Plasma levels rose rapidly into the nanogram range, peaked 2 h after MI, and then fell back to near baseline by day 1. In the myocardium, there was a similar early peak at 4 h, and there was a second peak 3 days after MI, before levels dropped to near baseline by day 7. Immunohistochemistry showed IL-6 production by myocytes, fibroblasts, and RCAECs during the first peak and by CD68 positive macrophages during the second (Supplemental Figure 6). Plasma concentration of sIL-6R peaked 1 day after MI before returning to baseline (Figure 2B), and the concentration of sgp130 was unchanged throughout the time course (Figure 2C).

Neutrophils rapidly infiltrated the myocardium and were significantly elevated by 4 h and peaked 1 day after MI, corresponding with the transient elevation in sIL-6R (Figure 2D). Classical monocyte numbers (Figure 2D) increased significantly also peaking 1 day after MI, but there was no significant change in the numbers of nonclassical monocytes or lymphocytes (Figure 2D).

There was a significant increase in numbers of 2 macrophage populations, major histocompatibility complex (MHC) II^pos^ and MHC-II^neg^, 3 days after MI, corresponding with the second peak in IL-6. This was most dramatic in the MHC-II^neg^ subset, which increased ∼20-fold from baseline (Figure 2D). This subset also had the highest surface CD11b expression (Figure 2E) and intracellular IL-6 production (Supplemental Figure 7).

These data informed the design of the subsequent experiments. First, they highlight 4 h, 1 day, and 3 days after MI as key time points in the inflammatory response that may be modulated by targeting IL-6. Second, for therapeutic experiments, they provided a rationale for targeting IL-6 at reperfusion and for at least 7 days thereafter. The design of these experiments is outlined in Supplemental Table 4.

### Anti–IL-6-Ab and sgp130Fc have differential effects on inflammation after reperfused MI

**Figure 3:**
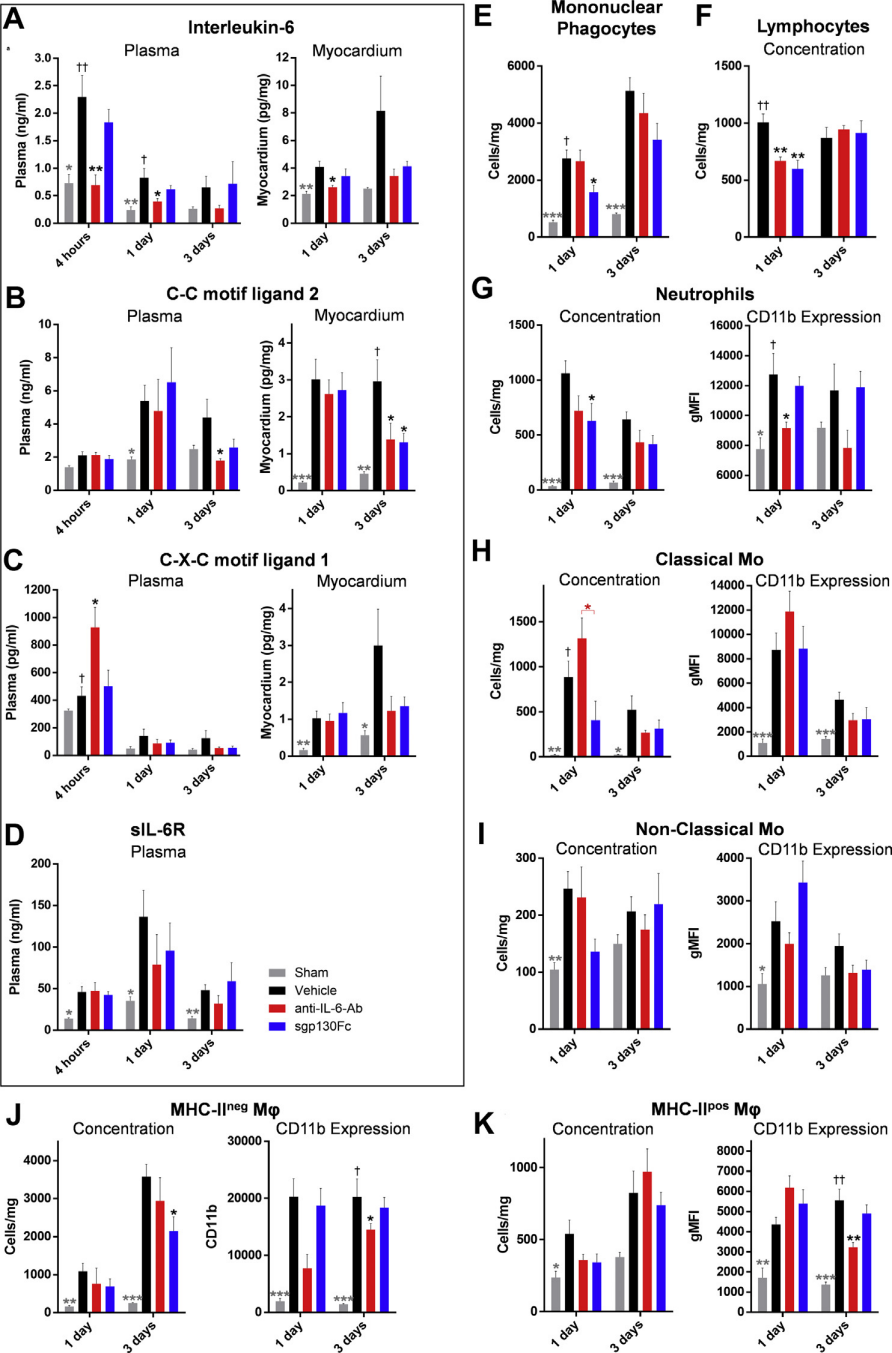
Effect of Anti–IL-6-Ab and sgp130Fc on Soluble Inflammatory Mediators and Leukocytes After MI With Reperfusion Rats were subjected to surgical myocardial infarction with 50 min of ischemia before reperfusion. One minute before reperfusion, vehicle (phosphate-buffered saline solution [PBS]), anti–IL-6-Ab (0.1 μg/mg, second dose intraperitoneally 3 days after MI), or sgp130Fc (0.5 μg/mg) was administered intravenously in 1 ml PBS (n = 5–9/group). **(A to D)** Cytokines in plasma and supernate of myocardial digests were measured by means of enzyme-linked immunosorbent assay and enzyme immunoassay. **(E to K)** Cell concentrations and CD11b expression were measured by means of flow cytometry. Data presented as mean ± SEM. Statistical significance was tested by means of analysis of variance (ANOVA) with multiple comparisons (comparing drug groups with vehicle; **black asterisks and daggers**) or unpaired Student’s *t*-tests (comparing sham with vehicle controls; **gray asterisks and daggers**). Where ANOVA was significant but differences between vehicle and the drug groups were not, a post hoc unpaired Student’s *t*-test was performed between the two drug groups **(red asterisk)**. Multiplicity-adjusted **(asterisks)** and ANOVA **(daggers)** p < 0.05 was considered to be significant. *∗*†p<0.05; ∗∗††p<0.01; ∗∗∗†††p<0.001. Ab = antibody; MHC = major histocompatibility complex; Mo = monocytes; other abbreviations as in Figures 1 and 2.

Anti–IL-6-Ab, but not sgp130Fc, reduced IL-6 concentrations in the plasma and myocardium (Figure 3A). Both drugs, however, significantly reduced myocardial CCL2 concentration 3 days after MI (Figure 3B), whereas only anti–IL-6-Ab increased C-X-C motif ligand 1 (CXCL1) concentration 4 h after MI (Figure 3C).

Neither drug altered plasma sIL-6R concentration (Figure 3D) or other soluble mediators, including the acute-phase proteins alpha-2-macroglobulin and alpha-1-acid glycoprotein (Supplemental Figure 8).

Relative to vehicle control, total mononuclear phagocyte (Figure 3E) and neutrophil (Figure 3G) counts were both reduced in the sgp130Fc group, and lymphocytes (Figure 3F) were reduced in both drugs groups 1 day after MI (Figure 3F). Crucially, at this time point the number of classic monocytes was significantly higher in the anti–IL-6-Ab group compared with the sgp130Fc group (Figure 3H). Neither drug had an effect on nonclassical monocytes (Figure 3I). Similarly, 3 days after MI, the number of highly inflammatory MHC-II^neg^ macrophages was significantly reduced only in the sgp130Fc group (Figure 3J). In blood, neither drug altered leukocyte numbers (Supplemental Figure 8G).

Interestingly, only anti–IL-6-Ab had a significant effect on leukocyte CD11b expression, suppressing it on neutrophils (1 day) (Figure 3G) and both macrophage subtypes (3 days) (Figures 3J and 3K), suggesting that this effect is classic signaling dependent.

**Figure 4:**
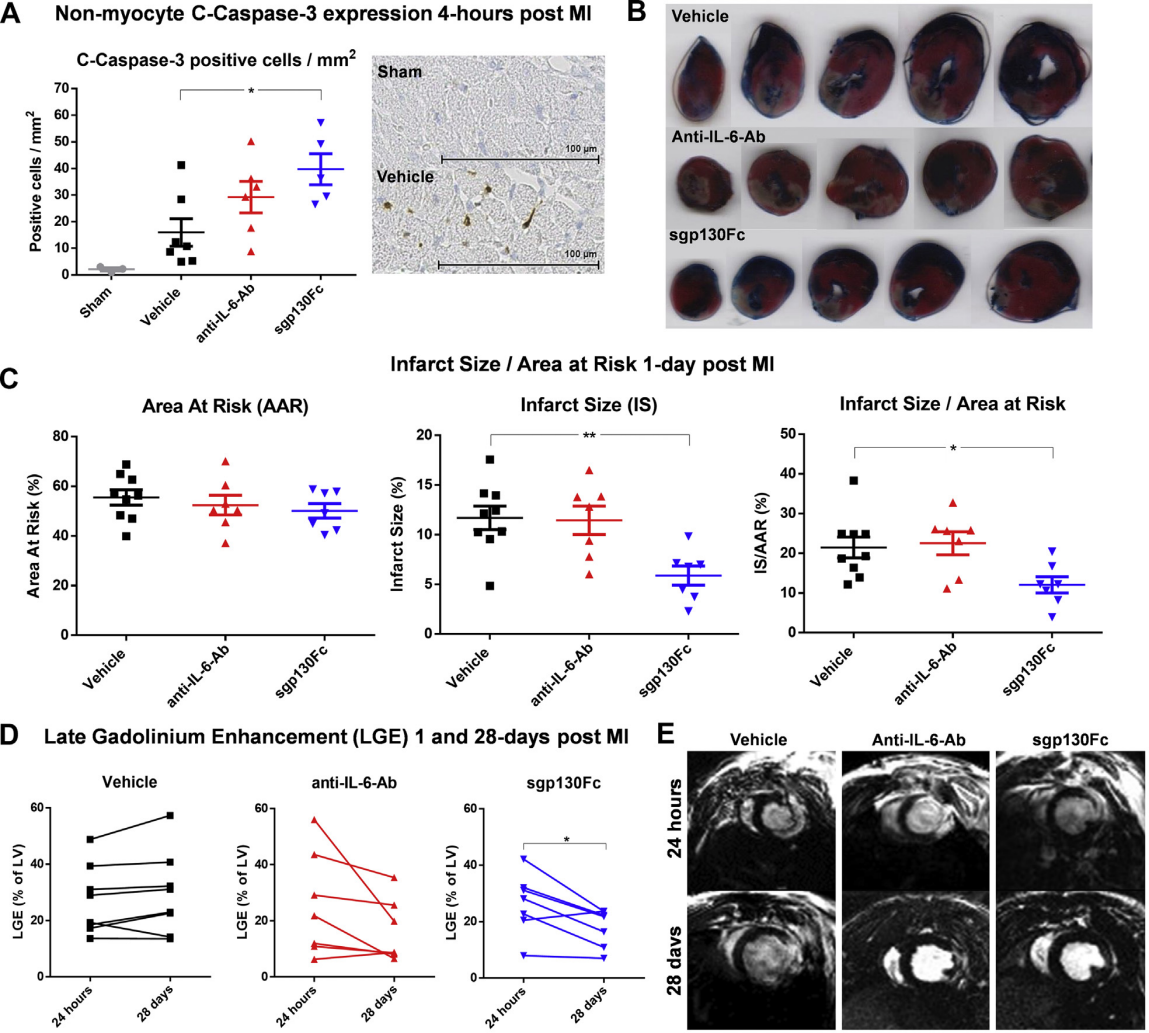
The Effect of Anti–IL-6-Ab and sgp130Fc on Infarct Size, c-Caspase-3 Expression, and LGE After MI With Reperfusion Rats were subjected to surgical myocardial infarction with 50 min ischemia prior to reperfusion. 1-min prior to reperfusion, vehicle (phosphate-buffered saline solution [PBS]), anti–IL-6-Ab (0.1 μg/mg, second dose intraperitoneally 3 days after MI) or sgp130Fc (0.5 μg/mg) were administered intravenously in 1 mL PBS (n = 7–9/group). **(A)** Hearts were excised 4 h after MI and stained with an anti–c-caspase-3 antibody. Nonmyocytes positive for c-caspase-3 were manually counted in a 4-mm^2^ transmural grid sited over the infarct area and expressed as positive cells/mm^2^ (n = 5–7/group). **(B, C)** Area at risk and infarct size were measured histologically by staining with 2,3,5-triphenyl-tetrazolium-chloride and Evans Blue and analyzed with the use of ImageJ 1 day after MI. **(D, E)** In further groups, electrocardiography-gated cardiac magnetic resonance–cine and gadolinium scans were performed 1 and 28 days after MI. LGE as percentage of total LV mass was calculated. Data presented as mean ± SEM. **(A, B)** Statistical significance tested with the use of analysis of variance with multiple comparisons (comparing the drug groups with vehicle) or unpaired Student's *t*-test (sham vs. vehicle). **(C)** Paired Student’s *t*-tests were performed to compare means of the 2 time points within each group. Multiplicity-adjusted p < 0.05 was considered to be significant. *∗*p < 0.05; ∗∗p < 0.01; ∗∗∗p < 0.001. c-Caspase-3, cleaved caspase-3; LGE = late gadolinium enhancement; LV = left ventricular; other abbreviations as in Figures 1 and 2.

Apoptosis likely contributed to reduction of inflammatory cells observed in the sgp130Fc group. In the sham group, c-caspase-3 staining of nonmyocytes was 2.08 ± 0.58 positive cells/mm^2^, and 4 h after MI in the vehicle group it was 15.93 ± 5.18 cells/mm^2^. However, in the anti–IL-6-Ab group this rose to 29.2 ± 5.92 cells/mm^2^ (p = 0.18 vs. vehicle) and in the sgp130Fc group to 39.72 ± 5.84 cells/mm^2^ (p = 0.019 vs. vehicle) (Figure 4A).

### sgp130Fc but not anti–IL-6-Ab reduces infarct size and LGE and preserves LVEF after reperfused MI

Having established the differential effects of the two drugs on inflammation, we next investigated their therapeutic efficacy.

Infarct size (IS) as a percentage of AAR (IS/AAR) was significantly reduced in the sgp130Fc group but not in the anti–IL-6-Ab group relative to vehicle controls. While IS/AAR in the vehicle group was 21.49 ± 2.63%, in the anti–IL-6-Ab group it was 22.55 ± 2.89% (p = 0.94 vs. vehicle) and in the sgp130Fc group 12.07 ± 2.04% (p = 0.031 vs. vehicle) (Figures 4B and 4C).

LGE from 1 to 28 days after MI was unchanged in the vehicle group (27.12 ± 4.34% vs. 29.35 ± 5.16%; p = 0.166), whereas there was a trend to reduction in the anti–IL-6-Ab group (25.7 ± 6.99% vs. 16.15 ± 4.21%; p = 0.099) and a significant reduction in the sgp130Fc group (26.4 ± 4.07% vs. 17.98 ± 2.53%; p = 0.0221) (Figures 4D and 4E).

Interestingly, 1 day after MI, LVEF was only significantly reduced in the anti–IL-6-Ab group compared with the naive group of rats: 58.06 ± 1.78% vs. 68.51 ± 1.82%, respectively (p = 0.015) (Table 1). However by 28 days, compared with the naive group, whose LVEF was 72.94 ± 1.59%, there were modest but significant reductions in both the vehicle group (62.58 ± 1.44%; p = 0.004) and the anti–IL-6-Ab group (63.34 ± 2.59%; p = 0.011) and a nonsignificant reduction in the sgp130Fc group (67.91 ± 2.88%; p = 0.25). Additional results from the CMR-cine imaging are included in Table 1.

**Table 1:** Cardiac Magnetic Resonance–Cine Results 1 Day and 28 Days After MI in Rats Administered Anti–IL-6-Ab or sgp130Fc

	Naive (n = 8)	Vehicle (n = 8)	Anti–IL-6-Ab (n = 7)	sgp130Fc (n = 7)	ANOVA p Value
End-diastolic volume, μl					
1 day	330.4 (275.2–385.7)	301.4 (278.1–324.7)	279.2 (242.1–316.3)	300 (247.2–352.8)	0.284
28 days	430.4 (350.8–510.1)	485.1 (429.8–540.4)	441.5 (386.9–496)	450.8 (361.9–539.6)	0.575
p value	0.001	<0.001	<0.001	0.001	–
End-systolic volume, μl					
1 day	105.6 (80.06–131.20)	112.6 (98.73–126.60)	116.1 (103.00–129.20)	105.1 (73.52–136.60)	0.803
28 days	117.8 (87.49–148.10)	181.3∗ (156.20–206.40)	163.8 (120.60–207.00)	147.4 (100.60–194.20)	0.032
p value	0.103	<0.001	0.027	0.009	–
Stroke volume, μl					
1 day	224.8 (191.6–258.0)	188.7 (167.7–209.8)	163.1∗ (133.1–193.1)	190.2 (144.8–235.5)	0.03
28 days	312.6 (257.1–368.1)	303.8 (265.0–342.6)	277.7 (246.5–308.8)	303.3 (244.6–362.1)	0.648
p value	<0.001	<0.001	<0.001	0.001	–
LVEF, %					
1 day	68.5 (64.2–72.8)	62.6 (58.5–66.6)	58.1∗ (53.7–62.4)	64.5 (54.7–74.3)	0.041
28 days	72.9 (69.2–76.7)	62.6† (59.2–66.0)	63.3∗ (57.0–69.7)	67.9 (60.9–75.0)	0.006
p value	0.033	0.985	0.034	0.153	–
Heart rate, beats/min					
1 day	403.1 (371.6–434.6)	401.5 (361.9–441.1)	390.7 (367.4–414.1)	391.4 (345.1–437.7)	0.905
28 days	325.1 (279.6–370.7)	356 (309.1–402.9)	358.4 (324.5–392.4)	317.1 (271.0–363.3)	0.298
p value	0.01	0.036	0.004	0.034	–
Cardiac output, ml/min					
1 day	90.3 (76.6–104.0)	75.6 (65.5–85.7)	63.7† (51.6–75.7)	73.6 (57.3–89.9)	0.016
28 days	101.7 (79.0–124.3)	107.9 (89.7–126.1)	99.8 (83.4–116.2)	95.3 (76.1–114.3)	0.742
p value	0.067	0.016	<0.001	0.031	–

Values are mean (95% confidence interval). Parameters from scans performed 1 day and 28 days after MI. The p values for paired Student’s *t*-tests performed within each group between the 2 time points are reported below the 2 time point values. Analysis of variance performed at each time point across all 4 groups and significance are reported in the righthand column (ANOVA p Value). Significance of individual pairwise comparisons with the naive group:

ANOVA = analysis of variance.

∗ p < 0.05;

† p < 0.01.

Collectively these data show that only sgp130Fc conferred a therapeutic benefit, reducing infarct size and preserving LVEF 28 days after MI.

## Discussion

This is the first study to investigate specific targeting of IL-6 trans-signaling in reperfused MI and to demonstrate the superior efficacy of this approach over panantagonism. Furthermore, this study provided novel insights into the temporal dynamics of IL-6 and the relative contribution of trans-signaling to the acute inflammatory response after MI.

### The temporal profile of IL-6 after reperfused MI

The biphasic temporal profile of IL-6 after MI with reperfusion has not previously been described. The early increase 4 h after MI is likely due to passive release from necrotic myocytes (22) as well as rapid transcription in viable myocytes, endothelial cells, and fibroblasts (8), all of which cells showed strong staining for IL-6 2 h after MI. A similar rapid rise has been described in humans (23). Circulating levels returned to baseline 1 day after MI, whereas in humans the levels may be raised for several days (24,25). The second peak at 3 days, which was restricted to the myocardium, appears to be largely due to the massive influx of mononuclear phagocytes, which stained strongly for IL-6. This was preceded by a rapid rise in sIL-6R concentration 1 day after MI, coinciding with peak neutrophil numbers which shed it to drive trans-signaling (13).

### Differential effects of anti–IL-6-Ab and sgp130Fc on IL-6 signaling

We used anti–IL-6-Ab to achieve panantagonism and sgp130Fc for exclusive trans-signaling blockade. The in vitro RCAEC data demonstrated that anti–IL-6-Ab is able to bind and neutralize IL-6, which prevents both classic and trans-signaling, as has been extensively described (13,15)**.** In contrast, sgp130Fc had no effect on free IL-6 in vitro, only blocking its action in the presence of sIL-6R and is therefore an exclusive trans-signaling antagonist. In vivo, only anti–IL-6-Ab, not sgp130Fc, reduced IL-6 concentration, providing direct evidence of their different effects on classic signaling. Although it is not possible to provide similar direct in vivo evidence for equipotent effects on trans-signaling, both reduced the trans-signaling–dependent chemokine CCL2 (4) to a similar degree, which is strongly suggestive that this was indeed the case. Thus, the two drugs achieved the intended differential effects on IL-6 signaling.

### The role of IL-6 classic and trans-signaling after reperfused MI

There were reductions in total mononuclear phagocytes and neutrophils 1 day after MI in those that received sgp130Fc, suggesting that this was a trans-signaling–dependent effect. There was no corresponding reduction in a specific chemokine to account for this observation. However, blockade of IL-6 trans-signaling reduces expression of endothelial adhesion molecules such as ICAM-1, which has been shown to attenuate cell trafficking (3,5). In addition, the reduced inflammatory cell numbers may also be due to increased apoptosis, given the increase in c-caspase-3 expression in nonmyocytes 4 h after MI. This is in keeping with data showing that IL-6 signaling has antiapoptotic effects on leukocytes (26).

Interestingly, 4 h after MI, anti–IL-6-Ab, but not sgp130Fc, increased the plasma concentration of CXCL1, suggesting that IL-6 classic signaling negatively regulates this chemokine, as previously described (27). CXCL1 drives monocyte trafficking (28) and this likely explains the increase in classical monocytes observed in the anti–IL-6-Ab group 1 day after MI.

That pan–IL-6 antagonism can increase CXCL family cytokines is supported by data from the Oslo NSTEMI TCZ trial. In a follow-up study, the group examined the effect of TCZ treatment on soluble mediators (25). Although they did not include CXCL1 in their measurements, the related CXCL chemokines CXCL8 (IL-8) and CXCL10 (interferon-γ–induced protein 10) were both elevated in the TCZ group (25).

Both drugs significantly reduced myocardial CCL2 3 days after MI. CCL2 is one of the primary chemokines responsible for monocyte trafficking in acute inflammation (4), and its reduction corresponded with the lower numbers of proinflammatory MHC-II^neg^ macrophages in the sgp130Fc group. The failure of anti–IL-6-Ab to reduce macrophage numbers to the same extent is likely a result of the early increases in CXCL1 and classical monocyte numbers.

In summary, we demonstrated that proinflammatory effects of IL-6 after MI are trans-signaling dependent, whereas classic signaling has antiinflammatory effects. Therefore, specific targeting of IL-6 trans-signaling had more pronounced antiinflammatory effects than pan-blockade (the proposed mechanism is shown in Supplemental Figure 9).

### Selective interleukin-6 trans-signaling blockade is more effective than panantagonism in reperfused MI

Only sgp130Fc, not anti–IL-6-Ab, reduced infarct size. This effect may be the result of its greater antiinflammatory effects compared with anti–IL-6-Ab. Neutrophils and mononuclear phagocytes, both of which were reduced in the sgp130Fc group, have been implicated in driving myocyte death through the release of reactive oxygen species and proteolytic enzymes (6) as well as in contributing to microvascular obstruction and the “no-reflow” syndrome (29). However, it is also possible that other mechanisms, such as direct effects on myocytes, contributed to this observation. For example, Smart et al. (30) demonstrated that IL-6 classic signaling protects isolated neonatal rat myocytes against ischemia-reperfusion injury in a phosphoinositide 3-kinase–dependent manner. Therefore, we speculate that this protection would be lost in the anti–IL-6-Ab group but preserved in the sgp130Fc group. This possibility warrants further study.

The early effects of sgp130Fc on inflammation and infarct size translated to a reduction in LGE as well as preserved LVEF 28 days after MI, demonstrating long-term beneficial effects of this strategy on cardiac remodeling and function. In contrast, LVEF was significantly reduced in the anti–IL-6-Ab group to the same extent as in the controls.

Our data regarding pan–IL-6 blockade reflect those of Hartman et al. (31), who are the only other group to pharmacologically antagonize IL-6 in an animal model of reperfused MI. They found that pan–IL-6 blockade with an anti–IL-6R MAb in a mouse model reduced LVEF (31). Therefore, current preclinical data do not support the hypothesis that pan–IL-6 blockade would be an effective therapy in reperfused MI.

However, in the phase 2 clinical trial of IL-6 receptor blockade with TCZ in NSTEMI, high-sensitivity Trop-T was reduced in the treatment group (9). Furthermore, results of ASSAIL-MI suggest improved myocardial salvage index with the use of TCZ after STEMI (12). Therefore, there may be interspecies variability in the effect of pan–IL-6 blockade in reperfused MI. One such difference may be the effect on neutrophils. In the NSTEMI study, a significant reduction in circulating neutrophil count was observed in the TCZ-treated group, which was proposed to mediate the beneficial effects of the drug (25). However, in the present study we did not observe a reduction in circulating neutrophil count in either drug group.

Although myocardial salvage index was reportedly improved by TCZ in the ASSAIL-MI trial, infarct size, Trop-T, N-terminal pro–B-type natriuretic peptide, and LV end-diastolic volume at 6 months were not (12). Therefore, while TCZ shows some promise in STEMI, our results suggest that selective IL-6 trans-signaling antagonism with sgp130Fc would have greater efficacy and should be investigated as a novel therapeutic in this setting. Under the trade name Olamkicept, sgp130Fc is currently undergoing phase II clinical trials in inflammatory bowel disease (32).

### Study limitations

While our study has provided evidence for the therapeutic potential of sgp130Fc in MI, some limitations are noteworthy. Our rat model resulted in a relatively small, largely apical infarct, which translated to a modest reduction in LVEF at 28 days of 10%. A larger infarct may have resulted in greater cardiac dysfunction and allowed better discrimination between groups at that time point.

In the characterization studies, we did not observe a rise in myocardial lymphocytes after MI, which are increasingly recognized to play an important role in myocardial injury and remodeling (33). However, our flow cytometry panel was not designed to identify individual lymphocyte subsets and therefore we can not exclude the possibility that there was an increase in specific cell types, such as CD4 T cells.

Finally, while we have demonstrated that sgp130Fc reduces infarct size and suggest that this is due to its anti-inflammatory effects, further work is required to discern the precise mechanisms which underpin this observation.

## Conclusions

We demonstrated for the first time that exclusive IL-6 trans-signaling antagonism with sgp130Fc is more effective than pan-blockade in reperfused MI. This approach attenuated inflammation, reduced infarct size, and preserved cardiac function. Given the pressing need to reduce the incidence of heart failure after MI, sgp130Fc warrants further investigation as a potential novel therapeutic in this setting.

Perspectives **COMPETENCY IN MEDICAL KNOWLEDGE:** The inflammatory cytokine IL-6 is associated with adverse outcomes after MI and is an emerging therapeutic target in coronary disease. IL-6 has 2 discrete signaling pathways: classic and trans. Trans-signaling mediates inflammation, while classic-signaling also has anti-inflammatory effects. Current approaches using anti–IL-6 antibodies block both pathways (panantagonism), whereas the novel recombinant protein sgp130Fc achieves selective IL-6 trans-signaling blockade. Using a rat model of reperfused MI, we demonstrated that sgp130Fc, but not anti–IL-6-Ab, attenuated neutrophil and macrophage infiltration into the myocardium, reduced infarct size, and preserved cardiac function 28 days after MI.

**TRANSLATIONAL OUTLOOK:** Targeting pan–IL-6 signaling with the monoclonal antibody tocilizumab has been shown to reduce C-reactive protein and troponin T in a phase 2 clinical trial of NSTEMI and to improve myocardial salvation index in STEMI. Our findings suggest that specific targeting of IL-6 trans-signaling in STEMI with sgp130Fc may be more effective that tocilizumab and should be investigated in the context of a clinical trial. Under the trade name Olamkicept, sgp130Fc is currently undergoing Phase-II clinical trials in inflammatory bowel disease.

## Funding Support and Author Disclosures

This work was supported by the Wellcome Trust (108735/Z/15/Z to Dr. George and 212937/Z/18/Z to Dr. Stuckey), the British Heart Foundation (FS/15/33/31608 and RM/17/1/33377 to Dr. Stuckey), the Medical Research Council (MR/R026416/1 to Dr. Stuckey), the National Institute for Health Research (Senior Investigator: Dr, Hingorani), and a King Scholarship of Malaysia (Ms. Jasmin). Dr. Woollard is an employee of AstraZeneca. No funding or support was received from AstraZeneca. All other authors have reported that they have no relationships relevant to the contents of this paper to disclose.

## References

[1] Ziaeian B., Fonarow G.C. (2016). Epidemiology and aetiology of heart failure. Nat Rev Cardiol.

[2] Fanola C.L., Morrow D.A., Cannon C.P. (2017). Interleukin-6 and the risk of adverse outcomes in patients after an acute coronary syndrome: observations from the SOLID-TIMI 52 (Stabilization of Plaque Using Darapladib-Thrombolysis in Myocardial Infarction 52) trial. J Am Heart Assoc.

[3] Chimen M., Yates C.M., McGettrick H.M. (2017). Monocyte subsets coregulate inflammatory responses by integrated signaling through TNF and IL-6 at the endothelial cell interface. J Immunol.

[4] Romano M., Sironi M., Toniatti C. (1997). Role of IL-6 and its soluble receptor in induction of chemokines and leukocyte recruitment. Immunity.

[5] Modur V., Li Y., Zimmerman G.A., Prescott S.M., McIntyre T.M. (1997). Retrograde inflammatory signaling from neutrophils to endothelial cells by soluble interleukin-6 receptor alpha. J Clin Invest.

[6] Entman M.L., Youker K., Shoji T. (1992). Neutrophil induced oxidative injury of cardiac myocytes. A compartmented system requiring CD11b/CD18-ICAM-1 adherence. J Clin Invest.

[7] Meléndez G.C., McLarty J.L., Levick S.P., Du Y., Janicki J.S., Brower G.L. (2010). Interleukin 6 mediates myocardial fibrosis, concentric hypertrophy, and diastolic dysfunction in rats. Hypertension.

[8] Wang J.-H., Zhao L., Pan X. (2016). Hypoxia-stimulated cardiac fibroblast production of IL-6 promotes myocardial fibrosis via the TGF-β1 signaling pathway. Lab Invest.

[9] Kleveland O., Kunszt G., Bratlie M. (2016). Effect of a single dose of the interleukin-6 receptor antagonist tocilizumab on inflammation and troponin T release in patients with non–ST-elevation myocardial infarction: a double-blind, randomized, placebo-controlled phase 2 trial. Eur Heart J.

[10] George M.J., Kleveland O., Garcia-Hernandez J. (2020). Novel insights into the effects of interleukin 6 antagonism in non–ST-segment-elevation myocardial infarction employing the SOMAscan proteomics platform. J Am Heart Assoc.

[11] Anstensrud A.K., Woxholt S., Sharma K. (2019). Rationale for the ASSAIL-MI-trial: a randomised controlled trial designed to assess the effect of tocilizumab on myocardial salvage in patients with acute ST-elevation myocardial infarction (STEMI). Open Heart.

[12] Dobkowski D. (September 2, 2020). IL-6 receptor inhibitor improves myocardial salvage in STEMI: ASSAIL-MI. Cardiology Today.

[13] Wolf J., Rose-John S., Garbers C. (2014). Interleukin-6 and its receptors: a highly regulated and dynamic system. Cytokine.

[14] Tilg H., Trehu E., Atkins M.B., Dinarello C.A., Mier J.W. (1994). Interleukin-6 (IL-6) as an antiinflammatory cytokine: induction of circulating IL-1 receptor antagonist and soluble tumor necrosis factor receptor p55. Blood.

[15] Garbers C., Heink S., Korn T., Rose-John S. (2018). Interleukin-6: designing specific therapeutics for a complex cytokine. Nat Rev Drug Discov.

[16] Jostock T., Müllberg J., Ozbek S. (2001). Soluble gp130 is the natural inhibitor of soluble interleukin-6 receptor transsignaling responses. Eur J Biochem.

[17] Schuett H., Oestreich R., Waetzig G.H. (2012). Transsignaling of interleukin-6 crucially contributes to atherosclerosis in mice. Arterioscler Thromb Vasc Biol.

[18] Carr C.A., Stuckey D.J., Tan J.J. (2011). Cardiosphere-derived cells improve function in the infarcted rat heart for at least 16 weeks—an MRI study. PLoS One.

[19] Barnett-Vanes A., Sharrock A., Birrell M.A., Rankin S. (2016). A single 9-colour flow cytometric method to characterise major leukocyte populations in the rat: validation in a model of LPS-induced pulmonary inflammation. PLoS One.

[20] Cheng C., Xu J.-M., Yu T. (2017). Neutralizing IL-6 reduces heart injury by decreasing nerve growth factor precursor in the heart and hypothalamus during rat cardiopulmonary bypass. Life Sci.

[21] Kobara M., Noda K., Kitamura M. (2010). Antibody against interleukin-6 receptor attenuates left ventricular remodelling after myocardial infarction in mice. Cardiovasc Res.

[22] Vanden Berghe T., Kalai M., Denecker G., Meeus A., Saelens X., Vandenabeele P. (2006). Necrosis is associated with IL-6 production but apoptosis is not. Cell Signal.

[23] Liebetrau C., Hoffmann J., Dörr O. (2015). Release kinetics of inflammatory biomarkers in a clinical model of acute myocardial infarction. Circ Res.

[24] Pannitteri G., Marino B., Campa P.P., Martucci R., Testa U., Peschle C. (1997). Interleukins 6 and 8 as mediators of acute phase response in acute myocardial infarction. Am J Cardiol.

[25] Kleveland O., Ueland T., Kunszt G. (2018). Interleukin-6 receptor inhibition with tocilizumab induces a selective and substantial increase in plasma IP-10 and MIP-1β in non–ST-elevation myocardial infarction. Int J Cardiol.

[26] Nakamura I., Omata Y., Naito M., Ito K. (2009). Blockade of interleukin 6 signaling induces marked neutropenia in patients with rheumatoid arthritis. J Rheumatol.

[27] Fielding C.A., McLoughlin R.M., McLeod L. (2008). IL-6 regulates neutrophil trafficking during acute inflammation via STAT3. J Immunol.

[28] Wang L., Zhang Y.-L., Lin Q.-Y. (2018). CXCL1-CXCR2 axis mediates angiotensin II–induced cardiac hypertrophy and remodelling through regulation of monocyte infiltration. Eur Heart J.

[29] Wang Z., Ren L., Liu N., Lei L., Ye H., Peng J. (2016). Association of monocyte count on admission with angiographic no-reflow after primary percutaneous coronary intervention in patients with ST-segment elevation myocardial infarction. Kardiol Pol.

[30] Smart N., Mojet M.H., Latchman D.S., Marber M.S., Duchen M.R., Heads R.J. (2006). IL-6 induces PI 3-kinase and nitric oxide-dependent protection and preserves mitochondrial function in cardiomyocytes. Cardiovasc Res.

[31] Hartman M.H.T., Vreeswijk-Baudoin I., Groot H.E. (2016). Inhibition of interleukin-6 receptor in a murine model of myocardial ischemia-reperfusion. PLoS One.

[32] Safety and Efficacy of TJ301 IV in Participants With Active Ulcerative Colitis. https://clinicaltrials.gov/ct2/show/NCT03235752.

[33] Hofmann U., Frantz S. (2015). Role of lymphocytes in myocardial injury, healing, and remodeling after myocardial infarction. Circ Res.

