# Supplementary material for: Selective Interleukin-6 Trans-Signaling Blockade Is More Effective Than Panantagonism in Reperfused Myocardial Infarction
Source: JACC Basic Transl Sci. 2021 Apr 7;6(5):431–43. doi: 10.1016/j.jacbts.2021.01.013 (PMC8165121; doi:10.1016/j.jacbts.2021.01.013)
Supplement: Supplemental Data [file mmc1.pdf]

Selective Interleukin-6 Trans-Signaling Blockade Is More Effective Than Panantagonism in Reperfused Myocardial Infarction

Supplemental Methods

Rat coronary artery endothelial cell culture

Rat coronary artery endothelial cells (RCAECs) were expanded in premade serum-containing media (Cell Applications, San Diego, California) supplemented with 10 µL/mL penicillin-streptomycin (10,000 U/mL; Gibco, Life Technologies, Carlsbad, California) in a sterile incubator (Galaxy R; Eppendorf, Hamburg, Germany) at 37°C with CO_2 at 5% and O_2 95%. Confluent RCAECs were used for experiments at P8 in 24-well plates (Corning Costar, Corning, New York), coated with 0.1% gelatine-based Attachment Factor (ThermoFisher, Waltham, Massachusetts). Serum-free medium (Cell Applications) was used for 24 hours before experiments. Confluent cells were imaged with the use of a NanoZoomer (Hamamatsu, Shizuoka Pref, Japan) to confirm endothelial cell morphology. RCAECs maintained their endothelial morphology characterized by a "cobblestone" monolayer of cells with diploid nuclei throughout the experiments.

Analgesia and euthanasia during surgery

Buprenorphine (0.05 mg/kg) was administered subcutaneously before recovery for analgesia. Euthanasia was performed by intraperitoneal administration of high-dose sodium pentobarbital (200 mg/kg) followed by exsanguination via cardiac puncture and removal of the heart.

Tissue preparation for flow cytometry and measurement of soluble mediators

Tissues from the rats were processed to measure the concentrations of soluble mediators as well as the number and activation status of inflammatory cells. This was done in blood (plasma) and the myocardium.

Blood was obtained immediately before excising the heart. Approximately 5–7 mL of blood was drawn via cardiac puncture and immediately added to 100 µL 0.5 mol/L EDTA (Sigma-Aldrich, St, Louis, Missouri) to prevent coagulation. The blood was spun at 650*g* for 10 minutes and subsequently the plasma was aspirated, aliquoted, and stored at −80°C. The cell pellet was further processed for flow cytometry (see below).

A protocol was developed to digest the myocardium. After cardiac puncture to obtain blood, the heart was excised. The aorta was cannulated and flushed to remove intracoronary blood. The great vessels were removed and then the whole heart was finely minced with sharp scissors. The tissue was weighed (Atilon balance; Acculab, Norwood, Massachusetts) and then placed in a 15-mL Falcon tube (Corning) together with 5 mL digest solution. The digest solution contained 200 µg/mL Liberase DL (Sigma-Aldrich) in phosphate-buffered saline solution (PBS) + $CaCl_2$ + $MgCl_2$ (ThermoFisher). Liberase DL contains collagenases I and II and a low concentration of dispase, a nonclostridial neutral protease. The heart tissue was then placed in an incubator at 37°C under constant agitation (200 rpm). After 45 minutes, the digest was removed and 100 µL 0.5 mol/L EDTA was added to chelate Ca^{2+} and stop the action of the enzymes. The digest was then passed through a 40-µm nylon strainer (Corning) before being spun at 650*g* for 5 minutes. The supernate was aspirated, aliquoted, and stored at −80°C for future

measurement of soluble mediators. The pellet was further processed for flow cytometry (see below).

Detailed methods for flow cytometry of inflammatory cells in the blood and myocardium

Protocol for staining surface markers

Cells were kept in 96-well plates throughout the staining process. Approximately 1×10^6 cells were resuspended in 100 µL flow-assisted cell sorting (FACS) buffer (5% fetal calf serum in PBS) together with antibodies. CD32 (Becton Dickinson [BD], Franklin Lakes, New Jersey) was used in all samples as an Fc blocker. Cells were stained at 4°C for 30 minutes. After spinning down (650*g*, 3 min), cells were resuspended in 100 µL wash buffer (2% fetal calf serum and 2 nmol/L EDTA in PBS) and then respun (650*g*, 3 min). Finally, cells were resuspended in 150 µL PBS and transferred to small FACS tubes containing 150 µL 1% formaldehyde (Sigma-Aldrich)—final concentration: 0.5% formaldehyde. Cells were analyzed with the use of LSR Fortessa (BD) with 5 lasers: ultraviolet (355 nm), violet (405 nm), blue (488 nm), green (561 nm) and red (640 nm). Gating for data acquisition was performed with the use of FACSDiva v.8.0.1 (BD). Flow cytometry data were analyzed with the use of FlowJo v.10.0.7 (BD). Cells were numerated with the use of CountBright Absolute Counting Beads (Molecular Probes, Eugene, Oregon). The gating strategy is illustrated in Supplemental Figure 2.

Wistar Kyoto CD68 Rats

CD68 is an established marker of mononuclear phagocytes in rats. However, because it is predominately expressed in the endosomal/lysosome compartment, there are no available antirat antibodies suitable for use in flow cytometry. Therefore, to corroborate that the gating strategy correctly identified mononuclear phagocytes, and conversely that the CD68 stains used in immunohistochemistry identified all subsets of macrophages, a novel strain of Wistar Kyoto rats expressing green fluorescent protein under the human CD68 promoter (GFP-CD68 rats) was used (Supplemental Figure 2). These rats were generated by means of Sleeping Beauty transposon germline transgenesis, as recently described (Turner-Stokes et al.; doi: 10.1681/ASN.2019121326). This approach confirmed that all monocyte and macrophage subsets strongly expressed CD68 whereas neutrophils and lymphocytes did not (Supplemental Figure 2 C).

Protocol for intracellular IL-6 measurement

Intracellular flow cytometry was performed to measure IL-6 production in individual cell types. Tissue was processed as described above, but after cell pellets had been isolated from blood and heart digests, the cells were resuspended in 5 mL Roswell Park Memorial Institute medium (ThermoFisher), supplemented with 0.4 µL/mL GolgiStop (Monensin; BD) and incubated at 37°C for 4 hours. This step was added to increase detectable levels of intracellular IL-6.

Cell surface staining was first performed with the use of a modified set of antibodies to accommodate the anti–IL-6 antibody on the AF488 channel. After surface staining, cells were spun at 350*g* for 5 minutes and then resuspended in 200 µL fixation buffer (Biolegend, San Diego, California) and left in the dark at 4°C for 20 minutes. After

spinning at 650*g* for 5 minutes, cells were washed with 200 µL Intracellular Staining Perm-Wash Buffer (Biolegend) before being resuspended in 100 µL Perm-Wash buffer containing goat polyclonal anti–IL-6 antibody (Ab) (IgG; R&D Systems, Minneapolis, Minnesota) or goat polyclonal IgG isotype control (R&D). After staining for 20 minutes in the dark at 4°C, cells were washed and spun before being resupended in 100 µL Perm-Wash buffer containing 10 µg/mL rabbit anti–goat IgG Ab conjugated to AF488 (Invitrogen, Carlsbad, California), After staining for 20 minutes in the dark at 4°C, the cells were washed and resuspended in FACS buffer. Samples were analyzed the same day on the LSR Fortessa (BD).

A dose-response curve of the antibody against the isotype control was performed to determine the optimal concentration of anti–IL-6-Ab to use. Based on this, the final concentration of Ab used was 5 µg/mL. Data from the intracellular IL-6 experiments are included in Supplemental Figure 7B.

Flow-assisted cell sorting

Flow-assisted cell sorting was performed with the use of FACS ARIA II (BD). Sorted cells were spun onto slides (Cytospin 2; Shandon, Cambridge, United Kingdom), stained with eosin and methylene blue (Kwick Diff; ThermoFisher), and imaged with the use of a NanoZoomer (Hamamatsu). Illustrative cell stains are included in Supplemental Figure 7A.

Immunohistochemistry

Hearts were fixed in 10% neutral-buffered formalin solution (Sigma-Aldrich) before being embedded in paraffin. Four-μm short-axis slices were taken at 3 to 4 evenly spaced intervals across the left ventricle. Immunohistochemistry was performed with the use of the fully automated Ventana Discovery XT instrument (Roche, Basel, Switzerland). After staining, slides were scanned with a Leica SCN400F scanner and visualized with the use of Leica software (Leica, Wetzlar, Germany). Representative images are included in Supplemental Figure 6.

ELISA and EIA kits used for measurement of soluble mediators

Kits that were used included:

- V-PLEX Proinflammatory Panel 2 (MSD, Kenilwort, New Jersey): IL-6, IL-1β, TNFα, IL-10, CXCL1, IFNγ, IL-4, IL-5, IL-10, IL-13 (plasma and heart digest).
- CCL2 Ultra-sensitive (MSD) (plasma and heart digest).
- sIL-6R (MyBiosource, San Diego, California) (plasma and heart digest).
- sgp130 (MyBiosource) (plasma).
- Acute-phase protein panel 1 kit (MSD): α2-macroglobulin and α1-acid glycoprotein (plasma).

Measurement of plasma levels of AF506 and determination of dosing frequency

To determine the dose of AF506, a minimum effective concentration (MEC) was estimated based on the characterization and RCAEC experiments. In the RCAEC experiments, a concentration of AF506 100× greater than that of IL-6 was necessary to fully antagonize its action, and the maximum plasma IL-6 concentration observed during

the characterization experiments was ~2.5 ng/mL. Therefore, the MEC for IL-6 antagonism was determined to be 100× this, that is, 0.25 µg/mL.

The circulating plasma volume of male rats can be estimated as ~4–5 mL/100 g. For a 200-g rat this equates to 8–10 mL. It was therefore calculated that a dose of 0.1 µg/g would result in a maximum plasma concentration of ~2–2.5 µg/mL, that is, 10× the minimum requirement.

To determine whether a single bolus of 0.1 µg/g AF506 would be sufficient to antagonize IL-6 for the desired time period (7 days), it was necessary to measure its concentration in vivo over time.

Given that AF506 is a goat polyclonal IgG, to achieve this an anti–goat IgG competitive inhibition enzyme immunoassay (EIA) kit was used (MyBioSource). AF506 reconstituted in naïve rat plasma was used as the control for the EIA rather than those supplied. Six serial 2-fold dilutions from 10 µg to 0.15625 µg were performed to produce a standard curve. These were analyzed with the use of the EIA kit according to the manufacturer's instructions. This experiment demonstrated that the EIA recognized AF506 with sufficient sensitivity to produce a hyperbolic standard curve with an R^2 of 0.994. Therefore, this assay could be used to measure the concentration of AF506 from plasma samples below that of the desired MEC of 0.25 µg/mL.

Ten rats underwent surgically induced MI with 50 minutes of ischemia before reperfusion, as described in the main article. 0.1 µg/g of AF506 was administered intravenously in 1 mL PBS (internal jugular vein) 1 minute before reperfusion. Five minutes after reperfusion, ~0.5 mL of blood was drawn from the internal jugular vein into

Eppendorff tubes with 10 µL 0.5 mol/L EDTA. The blood was spun (650*g*, 10 min) and the plasma frozen at −80°C.

Five rats were killed at 24 hours and the remaining 5 at 72 hours. Both groups had blood obtained via cardiac puncture, which was processed as described above.

Once all the samples had been collected, the plasma was analyzed with the anti–goat IgG EIA to determine the concentration over time (Supplemental Figure 3). Based on these measurements, a second dose of 0.1 µg/mg was administered intraperitoneally in 0.5 mL PBS 3 days after MI to ensure pan–IL-6 antagonism for at least 7 days after MI.

Detailed methods for cardiac magnetic resonance imaging

Rat preparation for CMR scanning

Rats were anesthetized in an infusion chamber under a mixture of 1.5%–2% isoflurane and O_2. They were then transferred to the cardiac magnetic resonance (CMR) scanning bed, which incorporates a rectal thermometer, heated water lines to maintain thermostasis (36.5–37°C), a respiration balloon to record respirations, 3 subcutaneous electrodes for recording electrocardiographic (ECG) signals, and a nose cone to deliver the anesthetic while the animal is in the scanner. The ECG, respiration, and temperature probes were connected to a physiological monitoring system (SA Instruments, Thane, India) to allow for image acquisition gating.

CMR image acquisition for measurement of left ventricular ejection fraction

A 9.4-T Varian MRI System was used (Palo Alto, Santa Clara, California) together with a 72-mm volume transmission coil with a 4-channel phased-array surface coil for detection. Electrical resonance of the radiofrequency coil was tuned and matched for optimal signal detection at the nuclear magnetic frequency of water using an external probe tuning device (505NV+; Morris Instruments, Sydney, Australia). The imaging system was controlled with the use of Varian VNMRJ software v.3.1 (Agilent, Santa Clara, California). This program performed imaging, shimming, and calibration protocols as well as basic reconstruction.

Cardiac- and respiratory-gated cinematic (cine) CMR imaging was performed in the true short-axis orientation and covered the whole left ventricle. CMR parameters: 1.5 mm slice thickness, 1.6 ms echo time, 5 ms repetition time, 15° pulse; 51.2 × 51.2 mm field of view; 128 × 128 matrix size; 400 × 400 × 1,500 µm voxel size; 25–35 frames per cardiac cycle, and 2 signal averages.

Gadolinium administration and image acquisition

Late gadolinium enhancement (LGE) scans were performed 15 minutes after intraperitoneal injection of 0.5 mmol/kg gadolinium (Magnevist; Bayer, Leverkusen, Germany). Images were acquired with the use of a multislice inversion recovery gradient recalled-echo sequence with single inversion time point at 300–400 ms. This approach provided the best contrast between healthy and infarcted myocardium. Ten to 12 short-axis slices were performed to cover the entire heart. CMR parameters: 3.04 ms echo time, 1.11 ms repetition time, 90° gradient-echo readout pulse, 1.5 mm slice thickness, 51.2 × 51.2 mm FOV, and 128 × 128 matrix size.

Cinematic CMR analysis

Images were analyzed off-line with the use of the Segment software (v.1.8; Medviso, Skane, Sweden). Semiautomatic segmentation of the stack of cine images was carried out, which identifies the left ventricular internal area (by identifying the interface between the blood pool and myocardium) and the epicardium. End-systolic and end-diastolic areas were measured for each slice and converted to volumes by multiplying by slice thickness (1.5 mm).

Stroke volume was calculated as end-diastolic volume − end systolic volume. Left ventricular ejection fraction for each slice was calculated as (end diastolic volumes − end systolic volume)/end-diastolic volume. Heart rate was measured by means of 3-lead ECG and used to calculate cardiac output as heart rate × stroke volume.

Left ventricular mass and late gadolinium enhancement analysis

Left ventricular mass and LGE images were analyzed with the use of ImageJ. Left ventricular mass was calculated as follows. First, manual segmentation of the myocardial area was performed. Second, this was converted to volume by multiplying by slice thickness (1.5 mm). Finally, volume was converted to mass by multiplying by the specific gravity of myocardium (1.05 g/mL).

LGE measurement was performed by using visual assessment and manual quantification. LGE was quantified manually by tracing around its perimeter. Enhancement on CMR images was presented as a percentage of the entire left ventricular myocardium.

Supplemental Figure 1: IL-6 Signaling Pathways and Pan Versus Trans-Signaling Antagonism

(A) Classic signaling is achieved by interleukin-6 binding to its membrane bound receptor (IL-6R) which associates with a dimer of membrane-bound glycoprotein 130 (gp130) to elicit intracellular signaling. **(B)** Trans-signaling is achieved by IL-6 binding to a soluble form of the IL-6 receptor (sIL-6R) and subsequently this IL-6/sIL-6R complex binds to ubiquitously expressed membrane-bound gp130. Alternatively, the IL-6/sIL-6R complex may bind to a soluble form of gp130, which prevents trans-signaling. **(C)** An antibody targeting IL-6 (or IL-6R) will prevent classic signaling as well as the formation of IL-6/sIL-6R complexes and therefore trans-signaling. **(D)** In contrast, sgp130Fc does not bind to free IL-6 and therefore does not prevent classic signaling. Neither does it prevent the formation of IL-6/sIL-6R complexes; however, once formed, it binds them with high affinity, preventing trans-signaling.

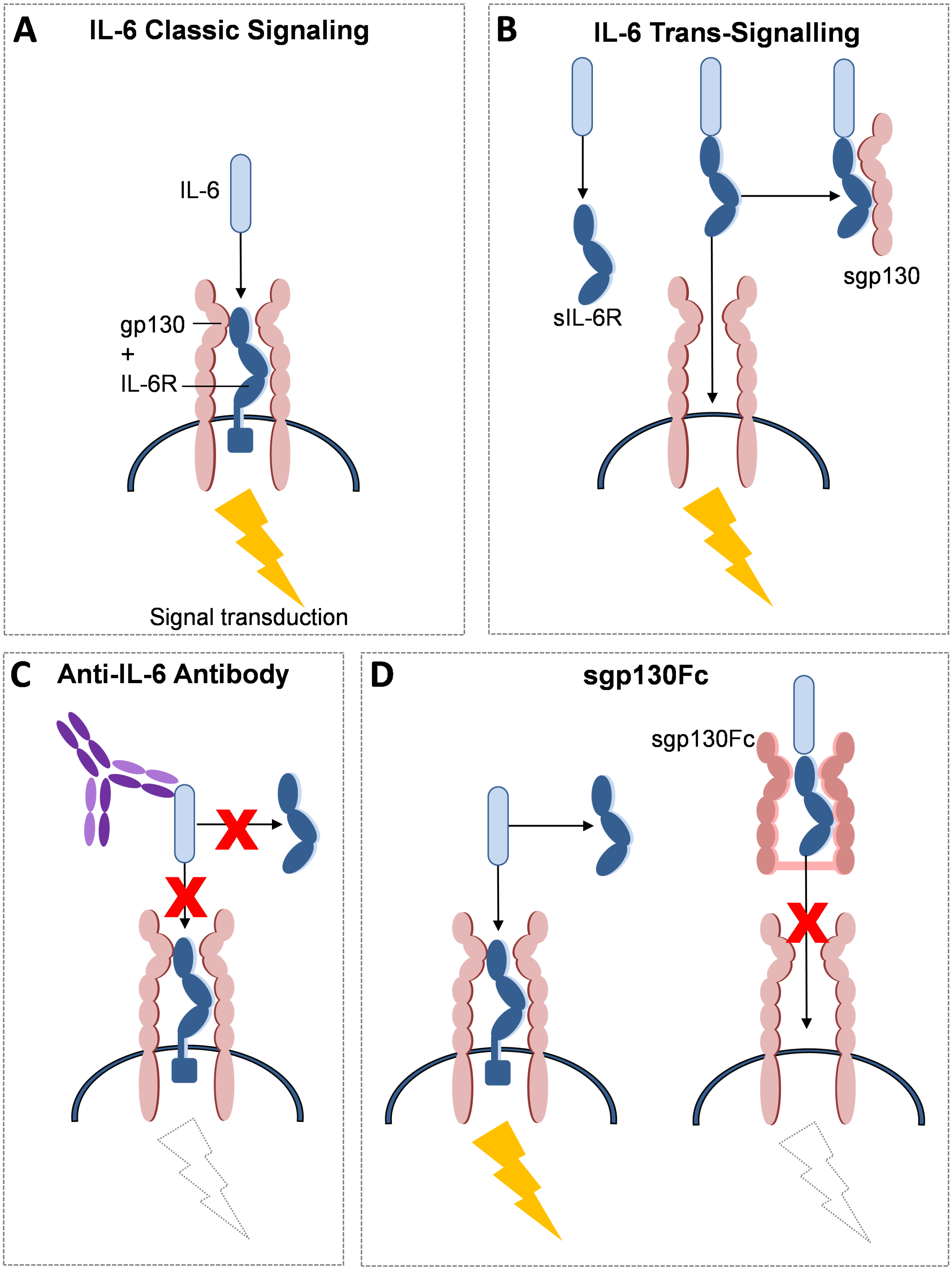
A
IL-6 Classic Signaling
IL-6
gp130
+
IL-6R
Signal transduction
B
IL-6 Trans-Signalling
sIL-6R
sgp130
C
Anti-IL-6 Antibody
D
sgp130Fc
sgp130Fc

Supplemental Figure 2: Blood and Myocardial Digest Gating Strategy to Identify Leukocytes by Means of Flow Cytometry

Cells were stained with the panel of antibodies listed in Supplementary Table 1. A similar approach was used to identify leukocytes in **(A)** the blood and **(B)** myocardial digests, with gating adjusted to differences in marker expression between the two tissues. Leukocytes were first identified with the use of CD45 **(Ai)**, with LiveDead stain included to aid identification in the myocardial digest **(Bi)**. Debris **(Aii, Bii)** and then doublets were excluded **(Aiii, Biii)**. Cells of the innate immune systems were identified as being positive for CD172α **(Aiv, Biv)**, whereas lymphocytes were negative. Next, MHC-II–positive cells were identified, representing dendritic cells in the blood **(Av)** and the MHC-II^{pos} macrophage population in the myocardium **(Bv)**. The MHC-II–negative cells were split into CD43-positive and -negative gates **(Avi, Bvi)**. The CD43-negative gate contained HIS48-positive classical monocytes **(Avii, Bvii)** and, in the myocardium, the MHC-II^{neg} macrophage population **(Bvii)**. The CD43-positive gate contained nonclassical monocytes and neutrophils, separated on the basis of side-scatter and HIS48 expression **(Avii, Bvii)**. **(C)** Representative color dot-plots shown from naïve blood and myocardium 4 hours after MI. Blood **(Ci)** and myocardial digest **(Cii)** from naïve GFP-CD68 rats were used to confirm the identify of mononuclear cells. Each cell type's expression of CD68 in the GFP channel was plotted against SSC-A. This showed that all monocyte and macrophage subsets strongly express CD68. GFP = green fluorescent protein; MHC = major histocompatibility complex; Mo = monocytes.

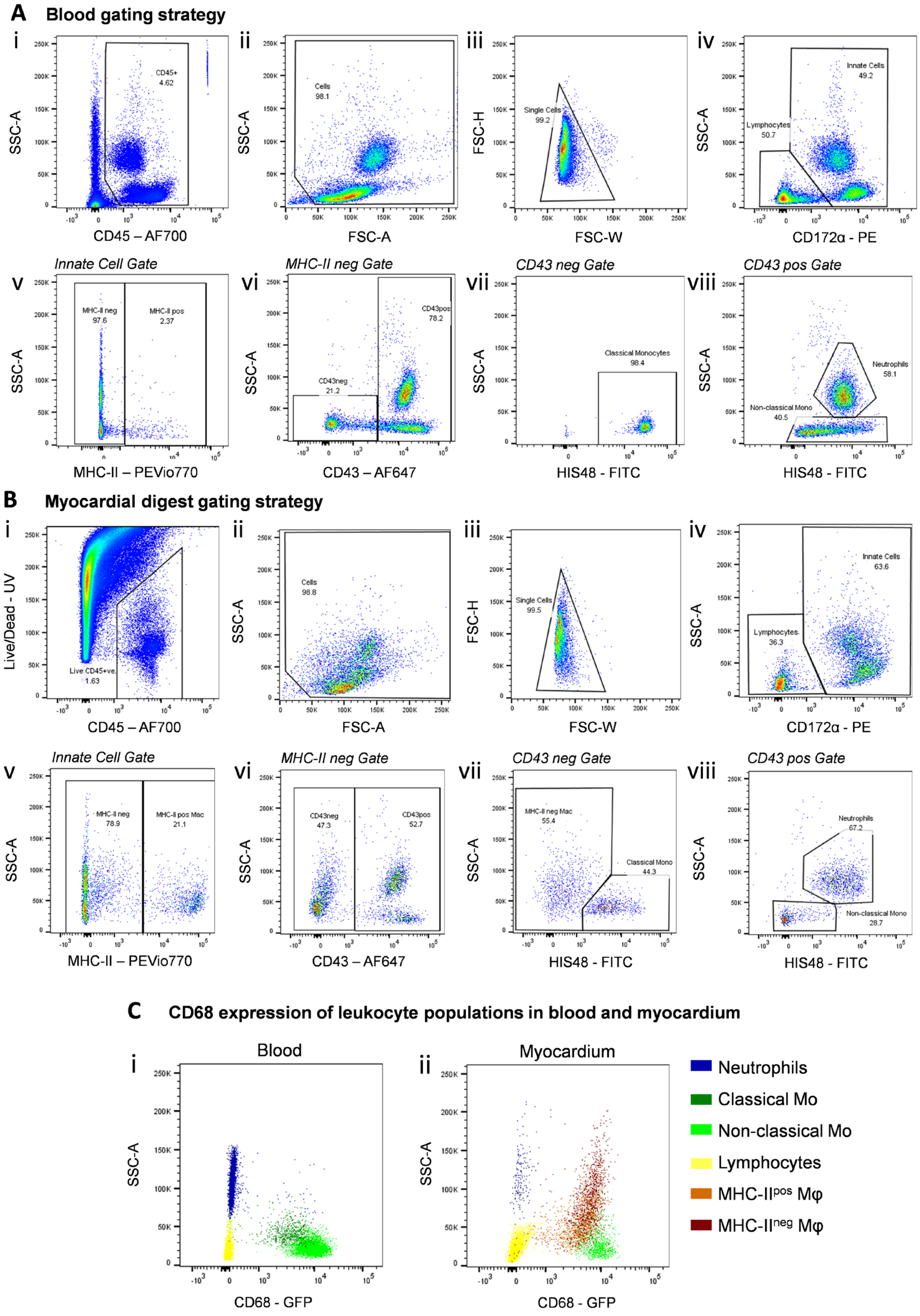
A Blood gating strategy
i
CD45+
4.62
SSC-A
CD45 – AF700
ii
Cells
98.1
SSC-A
FSC-A
iii
Single Cells
99.2
FSC-H
FSC-W
iv
Innate Cells
49.2
Lymphocytes
50.7
SSC-A
CD172α - PE
v
Innate Cell Gate
MHC-II neg
97.6
MHC-II pos
2.37
SSC-A
MHC-II – PEVio770
vi
MHC-II neg Gate
CD43pos
78.2
CD43neg
21.2
SSC-A
CD43 – AF647
vii
CD43 neg Gate
Classical Monocytes
98.4
SSC-A
HIS48 - FITC
viii
CD43 pos Gate
Neutrophils
58.1
Non-classical Mono
40.5
SSC-A
HIS48 - FITC
B Myocardial digest gating strategy
i
Live CD45+ve
1.63
Live/Dead - UV
CD45 – AF700
ii
Cells
98.8
SSC-A
FSC-A
iii
Single Cells
99.5
FSC-H
FSC-W
iv
Innate Cells
63.6
Lymphocytes
36.3
SSC-A
CD172α - PE
v
Innate Cell Gate
MHC-II neg
78.9
MHC-II pos Mac
21.1
SSC-A
MHC-II – PEVio770
vi
MHC-II neg Gate
CD43neg
47.3
CD43pos
52.7
SSC-A
CD43 – AF647
vii
CD43 neg Gate
MHC-II neg Mac
55.4
Classical Mono
44.3
SSC-A
HIS48 - FITC
viii
CD43 pos Gate
Neutrophils
67.2
Non-classical Mono
28.7
SSC-A
HIS48 - FITC
C CD68 expression of leukocyte populations in blood and myocardium
i
Blood
SSC-A
CD68 - GFP
ii
Myocardium
SSC-A
CD68 - GFP
Neutrophils
Classical Mo
Non-classical Mo
Lymphocytes
MHC-IIpos Mφ
MHC-IIneg Mφ

Supplemental Figure 3: Plasma Concentration of AF506 After a Single Dose Administered Before Reperfusion in Rat Model of MI

Ten rats underwent surgically induced MI with 50 minutes of ischemia before reperfusion. 0.1 µg/g of AF506 was administered in 1 mL phosphate-buffered saline solution intravenously 1 minute before reperfusion. Blood was taken 5 minutes after reperfusion and then rats were killed at 24 (n = 5) and 72 (n = 5) hours after MI. AF506 concentration was measured with an anti–goat IgG EIA. Data shown as mean ± SEM. A plasma concentration of 0.25 µg/mL (**dotted line**) was determined to be the minimum effective concentration (MEC) based on peak IL-6 plasma concentration and in vitro data (MEC = 100× Cmax of IL-6). On the basis of this data, a second dose of AF506 was given intraperitoneally 3 days after MI to ensure pan–IL-6 antagonism for the first 7 days after MI. MI = myocardial infarction.

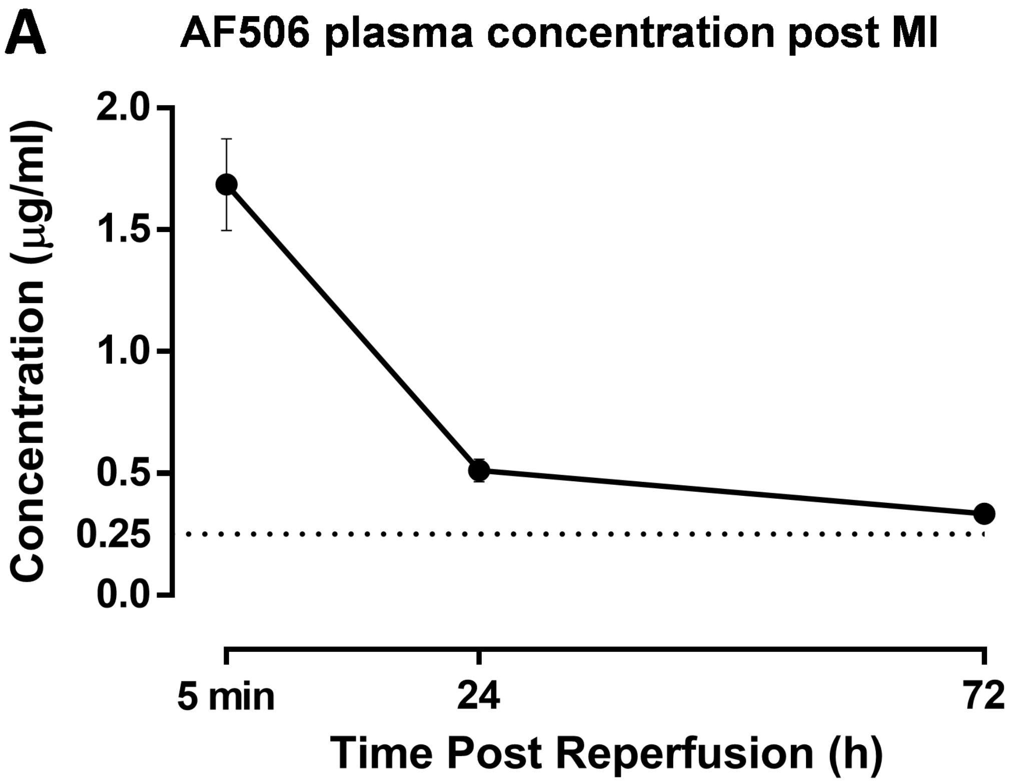
A
AF506 plasma concentration post MI
Concentration (μg/ml)
2.0
1.5
1.0
0.5
0.25
0.0
5 min
24
72
Time Post Reperfusion (h)

Supplemental Figure 4: Effect of Isotype Controls for sgp130Fc and AF506 on ICAM-1 Expression on RCAECs

Rat coronary artery endothelial cells (RCAECs) were seeded in 24-well plates at P8 and grown until confluent in serum-containing media. The cells were stimulated in serum-free media (total volume of 0.5 mL) for 16 hours. ICAM-1 expression was measured by means of flow cytometry. n = 3 (technical repeats), data shown as mean +SEM. Statistical significance was tested by means of analysis of variance with multiple comparisons (gray bars compared with unstimulated [#], black bars compared with 100 ng/mL IL-6 [*]). **$p < 0.01$; ###$p < 0.001$. ICAM-1 = intercellular adhesion molecule 1; IL-6 = interleukin-6.

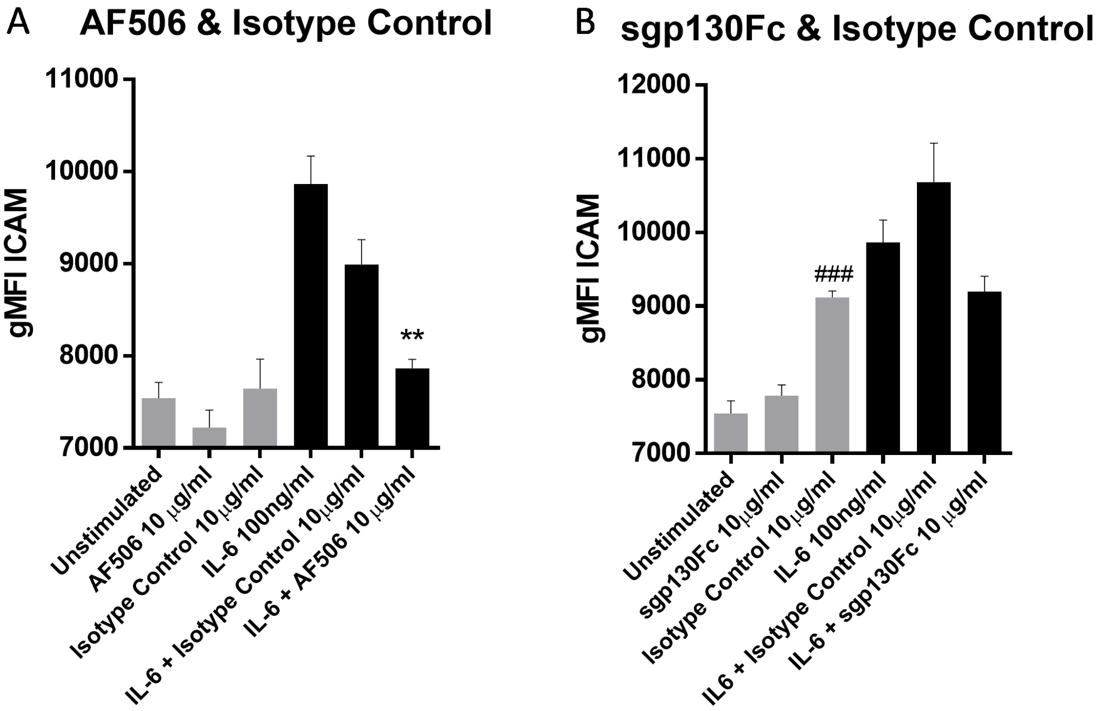
A
AF506 & Isotype Control
gMFI ICAM
11000
10000
9000
8000
7000
**
Unstimulated
AF506 10 μg/ml
Isotype Control 10μg/ml
IL-6 100ng/ml
IL-6 + Isotype Control 10μg/ml
IL-6 + AF506 10 μg/ml
B
sgp130Fc & Isotype Control
gMFI ICAM
12000
11000
10000
9000
8000
7000
###
Unstimulated
sgp130Fc 10μg/ml
Isotype Control 10μg/ml
IL-6 100ng/ml
IL6 + Isotype Control 10μg/ml
IL-6 + sgp130Fc 10 μg/ml

Supplemental Figure 5: In Vitro Antagonism of IL-6 Signaling With Anti–IL-6, anti–IL-6R Antibodies, and sgp130Fc

RCAECs were seeded in 24-well plates at P8 and grown in serum-containing medium. Once confluent, they were stimulated with 100 ng/mL IL-6 for 16 hours with increasing concentrations of **(A, B)** anti–IL-6 or **(C–E)** anti–IL-6R antibodies or **(F)** sgp130Fc alone. CCL2 was measured in the supernates by means of enzyme-linked immunosorbent assay, and surface ICAM-1 expression was quantified by means of flow cytometry. n = 3 (technical repeats), data shown as mean +SEM. Statistical significance was tested by means of analysis of variance with multiple comparisons (comparing each condition with 100 ng/mL IL-6; **black asterisks**) or unpaired *t* test (unstimulated vs. 100 ng/mL IL-6 A-E; **gray asterisks**). Multiplicity-adjusted $p < 0.05$ was considered to be significant. *$p < 0.05$; **$p < 0.01$; ***$p < 0.001$. CCL2 = C-C motif chemokine ligand; IL-6R = interleukin-6 receptor; other abbreviations as in Supplemental Figure 4.

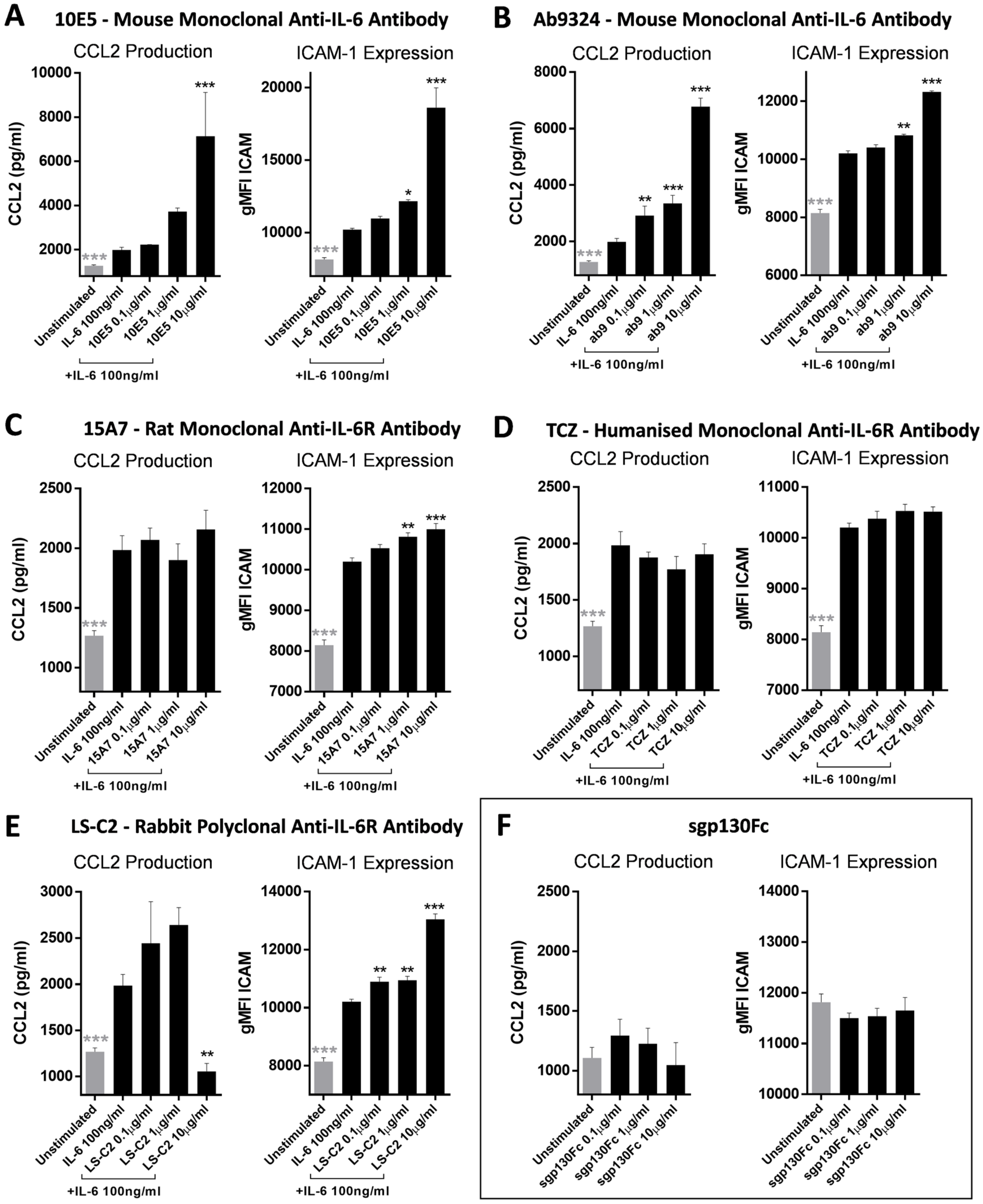
A
10E5 - Mouse Monoclonal Anti-IL-6 Antibody
CCL2 Production
ICAM-1 Expression
CCL2 (pg/ml)
gMFI ICAM
Unstimulated
IL-6 100ng/ml
10E5 0.1μg/ml
10E5 1μg/ml
10E5 10μg/ml
+IL-6 100ng/ml
B
Ab9324 - Mouse Monoclonal Anti-IL-6 Antibody
CCL2 Production
ICAM-1 Expression
ab9 0.1μg/ml
ab9 1μg/ml
ab9 10μg/ml
C
15A7 - Rat Monoclonal Anti-IL-6R Antibody
CCL2 Production
ICAM-1 Expression
15A7 0.1μg/ml
15A7 1μg/ml
15A7 10μg/ml
D
TCZ - Humanised Monoclonal Anti-IL-6R Antibody
CCL2 Production
ICAM-1 Expression
TCZ 0.1μg/ml
TCZ 1μg/ml
TCZ 10μg/ml
E
LS-C2 - Rabbit Polyclonal Anti-IL-6R Antibody
CCL2 Production
ICAM-1 Expression
LS-C2 0.1μg/ml
LS-C2 1μg/ml
LS-C2 10μg/ml
F
sgp130Fc
CCL2 Production
ICAM-1 Expression
sgp130Fc 0.1μg/ml
sgp130Fc 1μg/ml
sgp130Fc 10μg/ml

Supplemental Figure 6: IL-6 Immunohistochemistry in Naïve Heart and Hearts After MI

Hearts from **(A)** a naïve rat and rats **(B)** 2 hours and **(C)** 3 days after MI were fixed in formalin and then set in paraffin before being stained with an anti–IL-6 antibody. Myocardium shown at ×20 zoom and blood vessels at ×10 zoom. Sections from the naïve heart show no IL-6 staining of the myocardium **(A.1)** or endothelium **(A.2)**. Two hours after MI, there was significant IL-6 staining of myocytes **(B.1)** and endothelium **(B.3)** within the infarct zone **(B.1)** and nonmyocytes (predominately fibroblasts) within the border zone **(B)**. Three days after MI, there was some staining of myocytes within the core of the infarct **(C.1)**, but very little staining in the border zone **(C.2)** or endothelium **(C.3)**. However, there was significant staining within the inflammatory infiltrate **(C.4)**, which was predominately CD68 positive (CD68 stains not shown). MI = myocardial infarction.

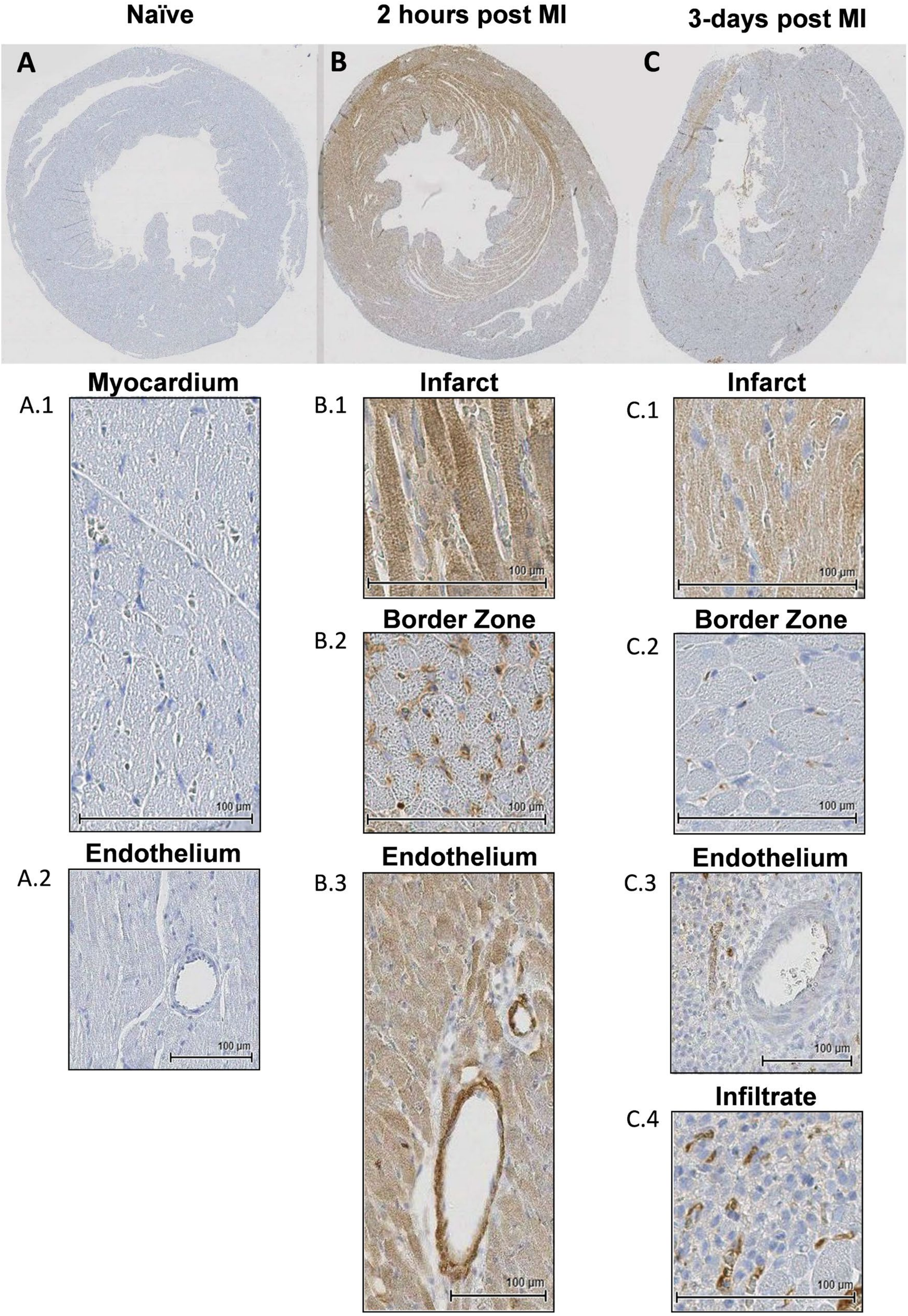

Naïve
2 hours post MI
3-days post MI
A
B
C
Myocardium
A.1
100 µm
Endothelium
A.2
100 µm
Infarct
B.1
100 µm
Border Zone
B.2
100 µm
Endothelium
B.3
100 µm
Infarct
C.1
100 µm
Border Zone
C.2
100 µm
Endothelium
C.3
100 µm
Infiltrate
C.4
100 µm

Supplemental Figure 7: Microscopy and Intracellular IL-6 Expression of Leukocytes

(A) Blood and heart digests were stained with the antibodies listed in Supplemental Table 1 and flow-assisted cell sorting was performed with gating performed per Supplemental Figure 2 to obtain populations of each cell type. Cells populations were cytospun onto slides before being stained with eosin and methylene blue. Slides were imaged with a NanoZoomer (Hamamatsu). Representative images shown at ×20 magnification. **(B)** Naïve rats (n = 4) or rats subjected to surgical myocardial infarction with 50 minutes of ischemia before reperfusion (n = 6) were included. Leukocytes from myocardial digests were identified, and intracellular IL-6 expression was quantified by means of flow cytometry. Statistical significance between the different cell types at each time point was tested by analysis of variance with multiple comparisons and between naïve and day 3 after myocardial infarction for each cell type with an unpaired Student *t*-test. Data presented as mean ± SEM. $p < 0.05$ was considered to be significant. *$p < 0.05$; **$p < 0.01$; ***$p < 0.001$. IL-6 = interleukin-6; MHC = major histocompatibility complex; Mo = monocytes.

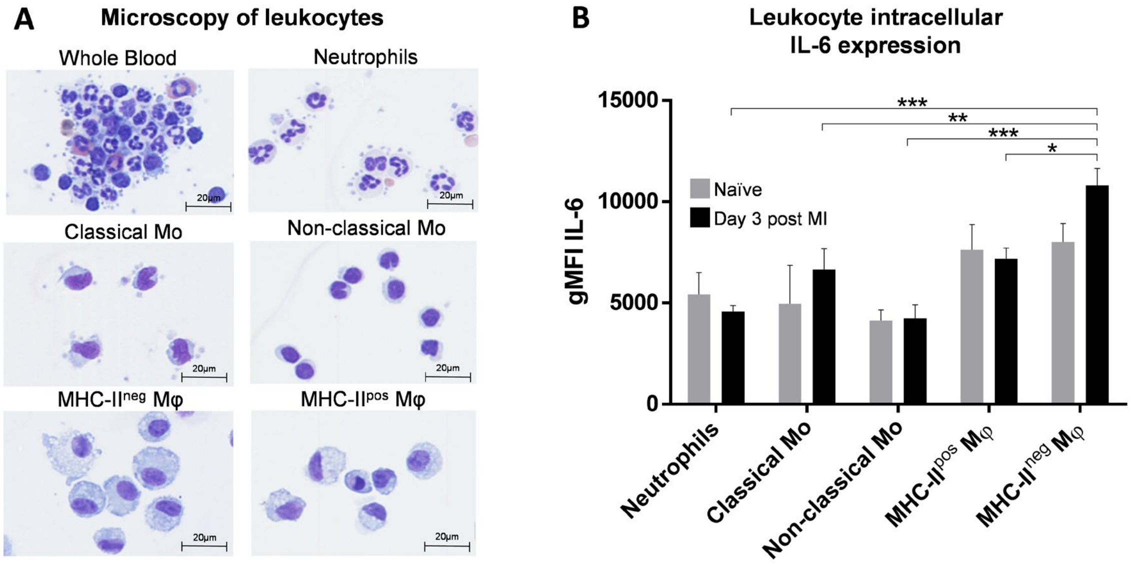
A
Microscopy of leukocytes
Whole Blood
Neutrophils
Classical Mo
Non-classical Mo
MHC-IIneg Mφ
MHC-IIpos Mφ
20µm
B
Leukocyte intracellular
IL-6 expression
gMFI IL-6
15000
10000
5000
0
***
**
***
*
Naïve
Day 3 post MI
Neutrophils
Classical Mo
Non-classical Mo
MHC-IIpos Mφ
MHC-IIneg Mφ

Supplemental Figure 8: Effect of Anti–IL-6-Ab and sgp130Fc on Soluble Inflammatory Mediators, Acute-Phase Proteins, and Blood Cell Counts After MI With Reperfusion

Rats were subjected to surgical myocardial infarction with 50 minutes of ischemia before reperfusion. One minute before reperfusion, vehicle (phosphate-buffered saline solution [PBS]), anti–IL-6-Ab (0.1 µg/mg, second dose intraperitoneally on day 3), or sgp130Fc (0.5 µg/mg) was administered intravenously in 1 mL PBS (n = 5–9/group). Cytokines and acute phase proteins in plasma ± supernate of myocardial digests were measured by means of enzyme-linked immunosorbent assay (ELISA) and enzyme immunoassay. Circulating leukocytes were identified and numerated by means of flow cytometry. Data presented as mean + SEM. Statistical significance was tested by analysis of variance with multiple comparisons (comparing drug groups with vehicle; **black asterisks**) or unpaired Student *t*-tests (comparing sham with vehicle control; **gray asterisks**). Multiplicity-adjusted $p < 0.05$ was considered to be significant. *$p < 0.05$; **$p < 0.01$; ***$p < 0.001$. A2M = alpha-2-macroglobulin; AGP = alpha-1-acid glycoprotein; IL = interleukin; Mo = monocytes.

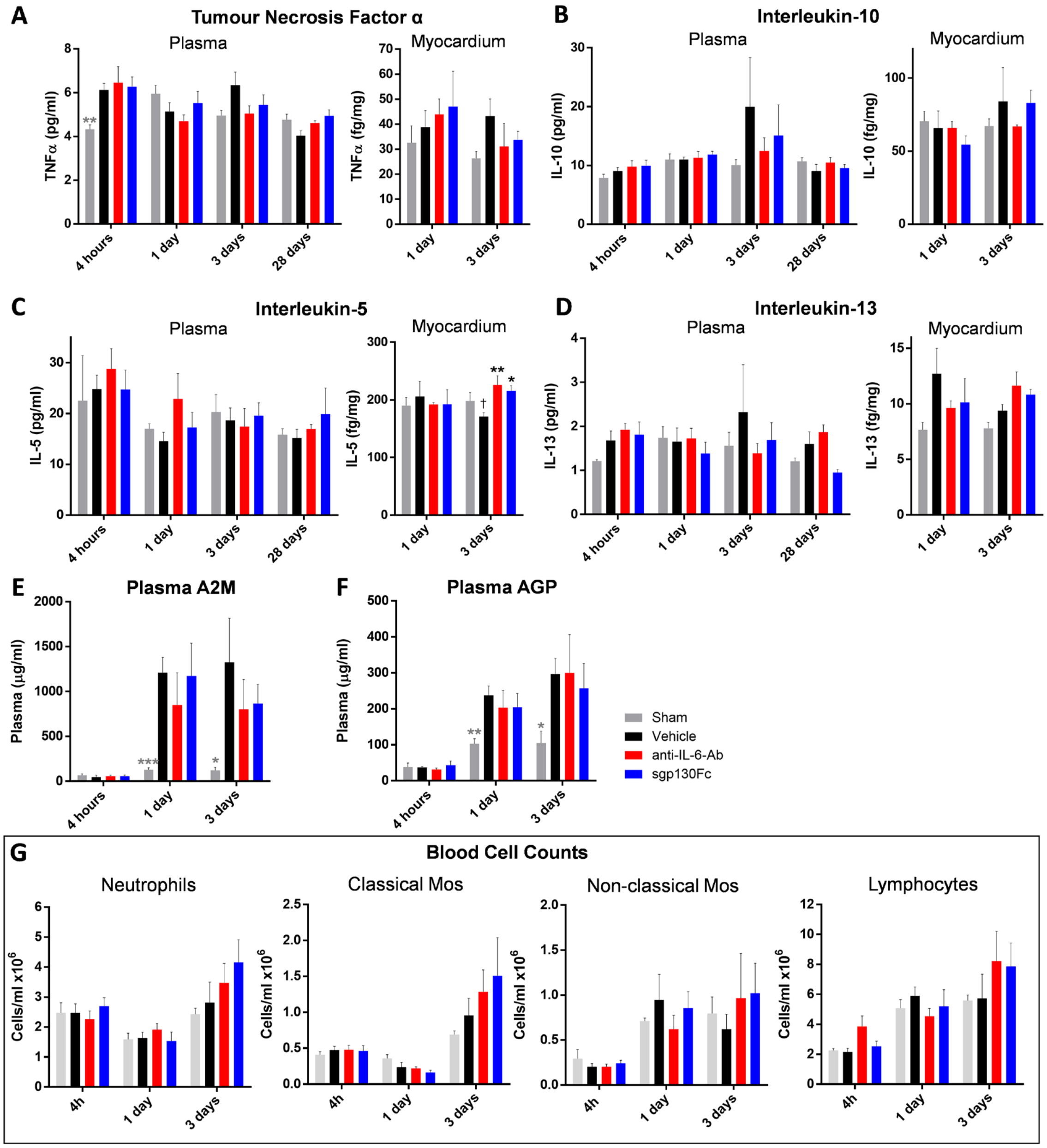
A
Tumour Necrosis Factor α
Plasma
Myocardium
TNFα (pg/ml)
TNFα (fg/mg)
B
Interleukin-10
Plasma
Myocardium
IL-10 (pg/ml)
IL-10 (fg/mg)
C
Interleukin-5
Plasma
Myocardium
IL-5 (pg/ml)
IL-5 (fg/mg)
D
Interleukin-13
Plasma
Myocardium
IL-13 (pg/ml)
IL-13 (fg/mg)
E
Plasma A2M
Plasma (µg/ml)
F
Plasma AGP
Plasma (µg/ml)
Sham
Vehicle
anti-IL-6-Ab
sgp130Fc
4 hours
1 day
3 days
28 days
G
Blood Cell Counts
Neutrophils
Classical Mos
Non-classical Mos
Lymphocytes
Cells/ml x10^6
4h
1 day
3 days

Supplemental Figure 9: Opposing Effects of IL-6 Classic and Trans-Signaling on Acute Inflammation

(A) Trans-signaling, mediated by the IL-6/sIL-6R complex binding to membrane-bound glycoprotein 130 facilitates the trafficking of classical monocytes and neutrophils by increasing endothelial expression of ICAM-1 and CCL2. Classic signaling suppresses cell trafficking by reducing production of CXCL1 by resident macrophages and dendritic cells, as well as ICAM-1 by endothelial cells. **(B)** Panantagonism with anti–IL-6-Ab blocks trans-signaling, resulting in reduced CCL2 and ICAM-1, but also blocks classic signaling, resulting in elevated CXCL1. The net effect is that there is a less significant reduction in the trafficking of inflammatory cells. **(C)** Exclusive trans-signaling blockade reduces CCL2 and ICAM-1 and maintains classic signaling suppression of CXCL1 production, resulting in a net reduction in inflammatory cell trafficking. Ab = antibody; CCL2 = chemokine C-C motif ligand; CMo = classical monocytes; CXCL1 = chemokine C-X-C motif ligand; ICAM-1 = intercellular adhesion molecule 1; IL-6 = interleukin-6; N = neutrophils.

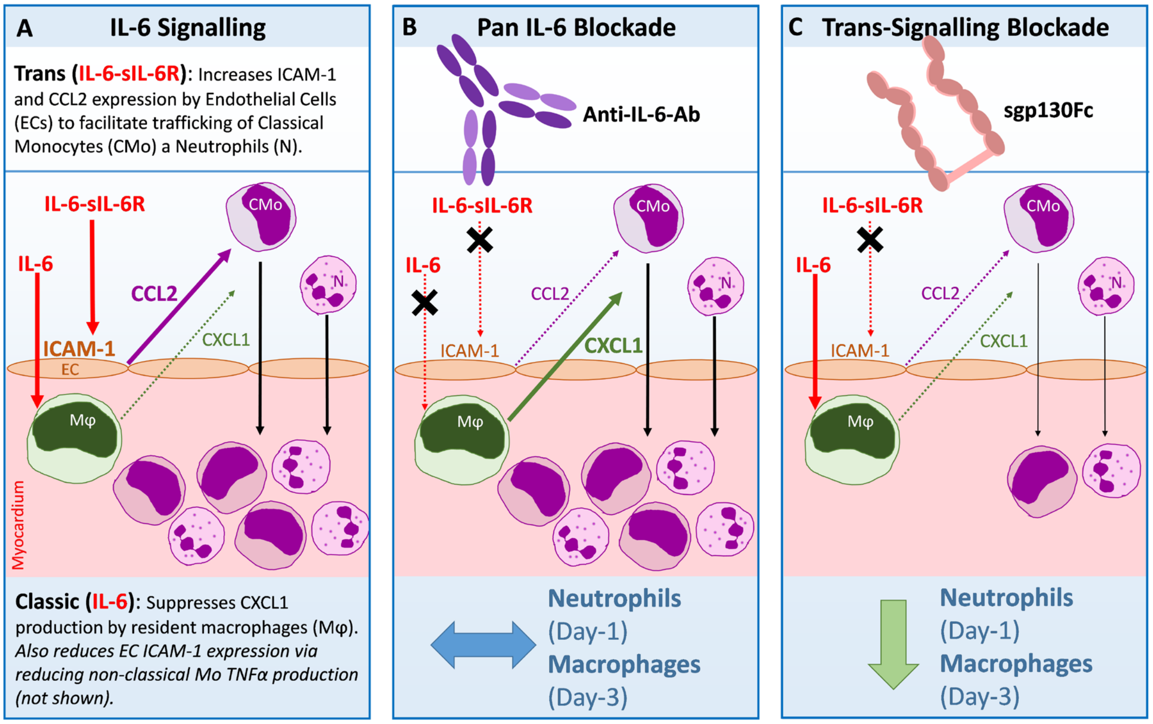
A IL-6 Signalling
Trans (IL-6-sIL-6R): Increases ICAM-1 and CCL2 expression by Endothelial Cells (ECs) to facilitate trafficking of Classical Monocytes (CMo) a Neutrophils (N).
IL-6-sIL-6R
IL-6
CCL2
CMo
N
ICAM-1
EC
CXCL1
Mφ
Myocardium
Classic (IL-6): Suppresses CXCL1 production by resident macrophages (Mφ). Also reduces EC ICAM-1 expression via reducing non-classical Mo TNFα production (not shown).
B Pan IL-6 Blockade
Anti-IL-6-Ab
IL-6-sIL-6R
IL-6
CCL2
CXCL1
ICAM-1
Neutrophils
(Day-1)
Macrophages
(Day-3)
C Trans-Signalling Blockade
sgp130Fc
IL-6-sIL-6R
IL-6
CCL2
CXCL1
ICAM-1
Neutrophils
(Day-1)
Macrophages
(Day-3)

Supplemental Table 1: Cell Surface Markers Used for Flow Cytometry

Marker	Fluorochrome	Manufacturer	Antibody	Concentration supplied, /mL	Volume per sample, µL
CD45	AF700	Biolegend	Mouse IgG1 k	0.5	1
CD172α	PE	Biolegend	Mouse IgG2a k	0.2	0.5
HIS48	FITC	BD	Mouse IgM k	0.5	0.5
CD43	AF647	Biolegend	Mouse IgG1 k	0.1	0.5
MHC-II	PE-Vio770	Miltenyi	Human IgG1	0.03	10
CD11b	V450	BD	Mouse IgA k	0.2	2.5
Live/dead	UV	ThermoFisher		–	200

Supplemental Table 2: Identification of Inflammatory Cells With the Use of Cell Surface Markers by Flow Cytometry

	Lymphocyte	Classical Monocyte	Nonclassical Monocyte	Neutrophil	MHC-II–Positive Macrophage	MHC-II–Negative Macrophage
CD172α	−	+	+	+	+	+
MHC-II	−/+	−	−	−	+	−
CD43	−/+	-	+	+	−	−
HIS48, blood	−	Hi	Low-Int	Int	−	−
HIS48, heart	−	+	−/+	+	−	−

MHC = major histocompatibility complex.

Supplemental Table 3: Anti–IL-6 and Anti–IL-6-R Antibodies Assessed With the RCAECs In Vitro Assay

Antibody Name	Host Species and Isotype	Supplier	Original Target Species	Used for Neutralization in the Rat previously?	References
Anti–IL-6 antibodies					
AF506	Goat poly IgG	R&D	Rat	Yes	1–6
10E5	Mouse mono IgG2	Insight	Rat	No	–
ab9324	Mouse mono IgG2	Abcam	Rat	No	–
Anti–IL-6 receptor antibodies					
15A7 (D7715A7)	Rat mono IgG2	Biolegend	Mouse	Yes	7–9
TCZ (Tocilizumab)	Human/mouse IgG1	Roche	Human	Yes	10–16
LS-C2 (LS-C299895)	Rabbit poly IgG	Source	Rat	No	–

References

1. Xu J, Kim HT, Ma Y, et al. Trauma and hemorrhage-induced acute hepatic insulin resistance: dominant role of tumor necrosis factor-alpha. Endocrinology 2008;149:2369–82.

2. Toth B, Yokoyama Y, Schwacha MG, et al. Insights into the role of interleukin-6 in the induction of hepatic injury after trauma-hemorrhagic shock. J Appl Physiol 2004;97:2184–9.

3. Tuna M, Erman T, Ildan F, et al. Effect of neutralization of rat IL-6 bioactivity on collateral blood supply from retrograde flow via cortical anastomoses in the rat central nervous system. Neurol Res 2002;24:405–8.

4. Cheng C, Xu J-M, Yu T. Neutralizing IL-6 reduces heart injury by decreasing nerve growth factor precursor in the heart and hypothalamus during rat cardiopulmonary bypass. Life Sci 2017;178:61–9.

5. Hashmat S, Rudemiller N, Lund H, et al. Interleukin-6 inhibition attenuates hypertension and associated renal damage in Dahl salt-sensitive rats. Am J Physiol Renal Physiol 2016;311:F555–61.

6. Farkas D, Alhussaini AA, Kraskauskas D, et al. Nuclear factor κB inhibition reduces lung vascular lumen obliteration in severe pulmonary hypertension in rats. Am J Respir Cell Mol Biol 2014;51:413–25.

7. McKinney WJ, Jaskot RH, Richards JH, et al. Cytokine mediation of ozone-induced pulmonary adaptation. Am J Respir Cell Mol Biol 1998;18:696–705.

8. Chen L-P, Zhang Q-H, Chen G, et al. Rapamycin inhibits cholangiocyte regeneration by blocking interleukin-6–induced activation of signal transducer and activator of transcription 3 after liver transplantation. Liver Transpl 2010;16:204–14.
9. König C, Morch E, Eitner A, et al. Involvement of spinal IL-6 trans-signaling in the induction of hyperexcitability of deep dorsal horn neurons by spinal tumor necrosis factor-alpha. J Neurosci 2016;36:9782–91.
10. Lin Y, Liu L, Jiang H, et al. Inhibition of interleukin-6 function attenuates the central sensitization and pain behavior induced by osteoarthritis. Eur J Pharmacol 2017;811:260–7.
11. Elcioğlu HK, Aslan E, Ahmad S, et al. Tocilizumab's effect on cognitive deficits induced by intracerebroventricular administration of streptozotocin in Alzheimer's model. Mol Cell Biochem 2016;420:21–8.
12. Wang S, Zhou J, Kang W, et al. Tocilizumab inhibits neuronal cell apoptosis and activates STAT3 in cerebral infarction rat model. Bosn J Basic Med Sci 2016;16:145–50.
13. Taskin MI, Gungor AC, Adali E, et al. A humanized anti–interleukin 6 receptor monoclonal antibody, tocilizumab, for the treatment of endometriosis in a rat model. Reprod Sci 2016;23:662–9.
14. Zhou R, Wu X, Wang Z, et al. Interleukin-6 enhances acid-induced apoptosis via upregulating acid-sensing ion channel 1a expression and function in rat articular chondrocytes. Int Immunopharmacol 2015;29:748–60.
15. Jiang P, Ling Q, Liu H, et al. Intracisternal administration of an interleukin-6 receptor antagonist attenuates surgery-induced cognitive impairment by inhibition of neuroinflammatory responses in aged rats. Exp Ther Med 2015;9:982–6.

16. Sari ES, Yazici A, Aksit H, et al. Inhibitory effect of sub-conjunctival tocilizumab on alkali burn induced corneal neovascularization in rats. Curr Eye Res 2015;40:48–55.

Supplemental Table 4: Groups of Rats Used in Therapeutic Experiments

	Time Point			
	4 Hours	1 Day	3 Days	28 Days
Blood/plasma	Cohort 1—soluble mediators and cells	Cohort 2—soluble mediators and cells	Cohort 3—soluble mediators and cells	
Heart digests		Cohort 2—soluble mediators and cells	Cohort 3—soluble mediators and cells	
Heart histology	Cohort 1—cleaved caspase-3	Cohort 4—infarct Size		
Imaging (CMR)		Cohort 5—LVEF and LGE		Cohort 5—LVEF and LGE
No. of rats per group	5–7	Mediators/cells: 5–8<br>Infarct size: 7–9	5–9	7–9

		LVEF and LGE: 7–9		

Five cohorts of rats were used for the therapeutic experiments. Within each cohort, there were groups for sham/naïve, vehicle control, anti–interleukin-6 antibody, and sgp130Fc. Cohort 1 was used for infarct size measurement 1 day after myocardial infarction (MI). Blood/plasma from cohorts 2–4 were used for measures of inflammation and hearts for immunohistochemistry (4 hours after MI) or for digests (1 day and 3 days after MI). Cohort 5 was used for cardiac magnetic resonance imaging (CMR) 1 day and 28 days after MI.

LGE = late gadolinium enhancement; LVEF = left ventricular ejection fraction.
